# Dynamic acylome reveals metabolite driven modifications in *Syntrophomonas wolfei*

**DOI:** 10.3389/fmicb.2022.1018220

**Published:** 2022-11-07

**Authors:** Janine Y. Fu, John M. Muroski, Mark A. Arbing, Jessica A. Salguero, Neil Q. Wofford, Michael J. McInerney, Robert P. Gunsalus, Joseph A. Loo, Rachel R. Ogorzalek Loo

**Affiliations:** ^1^Department of Chemistry and Biochemistry, University of California, Los Angeles, CA, United States; ^2^UCLA-DOE Institute, University of California, Los Angeles, CA, United States; ^3^Department of Microbiology and Plant Biology, University of Oklahoma, Norman, OK, United States; ^4^Department of Microbiology, Immunology and Molecular Genetics, University of California, Los Angeles, CA, United States; ^5^UCLA Molecular Biology Institute, University of California, Los Angeles, CA, United States; ^6^Department of Biological Chemistry, David Geffen School of Medicine, University of California, Los Angeles, CA, United States

**Keywords:** syntrophy, proteomics, mass spectrometry, post-translational modifications, lysine acylation, *Syntrophomonas wolfei*

## Abstract

*Syntrophomonas wolfei* is an anaerobic syntrophic microbe that degrades short-chain fatty acids to acetate, hydrogen, and/or formate. This thermodynamically unfavorable process proceeds through a series of reactive acyl-Coenzyme A species (RACS). In other prokaryotic and eukaryotic systems, the production of intrinsically reactive metabolites correlates with acyl-lysine modifications, which have been shown to play a significant role in metabolic processes. Analogous studies with syntrophic bacteria, however, are relatively unexplored and we hypothesized that highly abundant acylations could exist in *S. wolfei* proteins, corresponding to the RACS derived from degrading fatty acids. Here, by mass spectrometry-based proteomics (LC–MS/MS), we characterize and compare acylome profiles of two *S. wolfei* subspecies grown on different carbon substrates. Because modified *S. wolfei* proteins are sufficiently abundant to analyze post-translational modifications (PTMs) without antibody enrichment, we could identify types of acylations comprehensively, observing six types (acetyl-, butyryl-, *3-*hydroxybutyryl-, crotonyl-, valeryl-, and hexanyl-lysine), two of which have not been reported in any system previously. All of the acyl-PTMs identified correspond directly to RACS in fatty acid degradation pathways. A total of 369 sites of modification were identified on 237 proteins. Structural studies and *in vitro* acylation assays of a heavily modified enzyme, acetyl-CoA transferase, provided insight on the potential impact of these acyl-protein modifications. The extensive changes in acylation-type, abundance, and modification sites with carbon substrate suggest that protein acylation by RACS may be an important regulator of syntrophy.

## Introduction

Microbial syntrophy is an important component of the global carbon cycle. Recycling carbon anaerobically requires cooperation and cross-feeding between different microbial species to decompose large biopolymers initially into sugars, amino acids, and aromatic acids, which are then fermented to longer chain fatty acids and alcohols (McInerney et al., 2009). Syntrophic bacteria play a key role in anaerobic decomposition by degrading the fatty acids and alcohols produced by fermentative bacteria to acetate, hydrogen, formate, and CO_2_, which are substrates for their methanogenic partners (Schink, 1997). Metabolic cooperation during syntrophic metabolism is essential due to the unfavorable thermodynamics of these reactions if syntrophic end products such as hydrogen and/or formate accumulate. Under standard conditions, the degradation of aliphatic and aromatic acid intermediates by syntrophs is endergonic and the associated free energy is highly dependent on entropy (McInerney and Beaty, 1988). In effect, the flux of compounds into and out of the system plays a large role in whether reactions will proceed forward spontaneously. By rapidly consuming the products of fatty acid degradation (hydrogen, formate, and acetate), methanogens keep the concentrations of these compounds low enough for syntrophic fatty acid degradation to become exergonic (McInerney and Beaty, 1988). The result is a tightly knit network of interacting microbial species that rely on one another to convert biopolymers to methane and carbon dioxide. A thorough understanding of the metabolic interactions converting organic carbon to methane, especially syntrophy, is key to understanding the fate of organic carbon in anaerobic environments (Amos and Mcinerney, 1989; McInerney et al., 2009; Stams et al., 2012; Boll et al., 2020).

*Syntrophomonas wolfei* is a syntrophic bacterium that is a leading model for examining how microbial communities degrade short-chain fatty acids (McInerney et al., 1981; Lorowitz et al., 1989). To metabolize saturated fatty acids, the organism must be co-cultivated with a hydrogen/formate-consumer; typically the model methanogen *Methanospirillum hungatei* has been used (McInerney et al., 1979, 1981). However *S. wolfei* can degrade unsaturated fatty acids, such as crotonate, in either pure or coculture (Beaty and McInerney, 1987; Amos and McInerney, 1990), thus enabling comparisons of how this bacterium shifts its carbon metabolism to adapt to changing carbon substrates. The ability to grow *S. wolfei* in pure and multispecies cultures has resulted in its use as a model for studying anaerobic, syntrophic butyrate degradation. *S. wolfei* utilizes β-oxidation to degrade short chain fatty acids, for which the *S. wolfei* subspecies *wolfei* strain Göttingen genome encodes many paralogs: nine acyl-CoA dehydrogenase genes, five enoyl-CoA hydratase genes, six *3-*hydroxyacyl-CoA dehydrogenase genes, and five acetyl-CoA acetyltransferase genes (Sieber et al., 2010). While the main enzymatic steps have been identified, many questions remain about substrate specificity and regulation of these paralogs. Proteomic evidence indicates that, remarkably, altering growth from axenic (cultivation as a single species) to syntrophic conditions (cultivation with *M. hungatei*) does not change protein abundance significantly (Schmidt et al., 2013; Sieber et al., 2015; Crable et al., 2016). Despite the consistency in enzyme constituents, it is evident that enzymatic catalysis rates do change with growth condition (Wofford et al., 1986; McInerney and Wofford, 1992). If these activity changes do not reflect shifts in cellular transcription or translation, we hypothesized that the enzymes themselves were impacted by post-translational modifications (PTMs) in a manner that correlates with the changing environment.

PTMs are present in all kingdoms of life and have a plethora of structures, physicochemical properties, and by extension, functions (Walsh and Jefferis, 2006). PTMs have long been attractive candidates for regulating various cellular functions and metabolic pathways; intracellular conditions can be sensed and directly alter proteins. One class of such PTMs is lysine acylation, a family of diverse modifications. Uniquely, lysine can be acylated either by enzyme-mediated reactions (Ali et al., 2018) or spontaneously by reactive metabolites like reactive acyl-Coenzyme A species (RACS) or acyl phosphates (Wagner and Payne, 2013; Wagner and Hirschey, 2014; Baeza et al., 2015). The resulting acylation events have been shown to both impede and initiate enzymatic activity (Gardner et al., 2006; Garrity et al., 2007). These PTMs have been identified across all domains of life (Verdin and Ott, 2015; Christensen et al., 2019; VanDrisse and Escalante-Semerena, 2019) and have conserved mechanisms of acyl-regulation (Greiss and Gartner, 2009; Sanders et al., 2010). In eukaryotes, these modifications are primarily found in the mitochondria and are implicated in a wide range of human metabolic-related diseases (Anderson and Hirschey, 2012; Ali et al., 2018; Baldensperger and Glomb, 2021). In prokaryotes, acyl-PTMs modify proteins involved in metabolism in addition to other biological processes such as transcription, chemotaxis, and protein stability (Bernal et al., 2014; Christensen et al., 2019; VanDrisse and Escalante-Semerena, 2019).

Protein lysine acylation by RACS can result directly from the flux through metabolic pathways that generate the reactive metabolites. These PTMs may record variations in carbon flux due to stress or changes in substrate use, providing a mechanism to regulate carbon flow (Schilling et al., 2015; Trub and Hirschey, 2018). Acyl-lysine modifications were characterized recently in *Syntrophus aciditrophicus* and shown to correspond directly to RACS generated from the syntroph’s degradation of benzoate (Muroski et al., 2022). Enzymes involved in degrading benzoate were not only modified by the respective acyl-CoA intermediate utilized or produced, but also by additional RACS generated by other enzymes in the pathway. The fact that active deacylases (sirtuins) were identified suggests that acylations impact syntrophic metabolism. *S. aciditrophicus* displayed acylation levels abundant enough to forego antibody enrichment strategies that are often used to study protein modifications in mass spectrometry-based proteomics analyses (Zhao and Jensen, 2009; Mertins et al., 2013) and the study raised the possibility that abundant acylations exist in other syntrophs like *S. wolfei*, that generate RACS during their metabolism. If acyl-PTMs are a feature of syntrophy, they might play an important role in metabolism, which would be indicated by changes in PTMs with carbon substrate.

Here, we use mass spectrometry-based proteomics to take a systems level approach to identify acyl-modifications in the *S. wolfei* proteome from cells cultivated on different environmental substrates. Without utilizing PTM-specific enrichment strategies, we demonstrate how the acylome profile changes with growth condition—qualitatively in the acyl groups that modify lysines and quantitatively in their abundance. In particular, enzymes involved in the β-oxidation pathway were found to be heavily decorated with a wide range of acyl groups, all of which are related to RACS found in fatty acid oxidation. Specific sites changed in both the type and abundance of modifications observed under different conditions. To probe the effect that PTMs may have on protein function, structural studies and *in vitro* acylation assays were performed on acetyl-CoA transferase, a β-oxidation pathway enzyme observed to be extensively modified.

## Materials and methods

### Culturing of cells

*Syntrophomonas wolfei* subspecies *wolfei* strain Göttingen (DSM 2245B; Lorowitz et al., 1989; hereafter referred to as *S. wolfei* Göttingen) cells were grown axenically with crotonate as the carbon substrate (McInerney et al., 1981), or in coculture with methanogenic partner, *Methanospirillum hungatei* JF1 (DSM864), on butyrate as previously described (McInerney et al., 1979). Cells were harvested anaerobically and then frozen and stored at −70°C until processed (Muroski et al., 2022). *Syntrophomonas wolfei* subspecies *methylbutyratica* strain 4J5T (JCM 14075) was grown in the presence of methanogenic partner *M. hungatei* as described above but using the substrates, butyrate, crotonate, 2-methylbutyrate, valerate or hexanoate. Analyses were performed on three biological replicates from each culture condition.

### Sample preparation

Cell pellets were suspended in 4.0% w/v ammonium lauryl sulfate, 0.1% w/v sodium deoxycholate, and 5 mM tris(2-carboxyethyl)phosphine in 100 mM ammonium bicarbonate (ABC) for lysis. Proteins were alkylated and digested using enhanced filter-assisted sample preparation (eFASP) as described by Erde et al. (2014). Briefly, the lysate was exchanged into buffer composed of 8 M urea, 0.1% w/v sodium deoxycholate, and 0.1% w/v *n*-octyl glucoside with a 10 kDa Microcon® ultrafiltration unit (Millipore). Proteins were alkylated with 17 mM iodoacetamide and digested overnight at 37°C with a 1:100 ratio of trypsin:protein. Detergents were extracted from the peptide-containing buffer into ethyl acetate. Peptides were dried and stored at −20°C until used in MS experiments or for offline fractionation.

### Mass spectrometry

Peptides were dried, resuspended in 0.1% acetic acid, desalted with STAGE tips (Rappsilber et al., 2007) fabricated from 3M Empore C18 solid phase extraction disks, and then re-dried. Resultant peptide-containing samples were resuspended in LC–MS injection buffer (3% acetonitrile and 0.1% formic acid) and analyzed on an Orbitrap Exploris^™^ 480 Mass Spectrometer (ThermoFisher Scientific) using liquid chromatography–tandem mass spectrometry (LC–MS/MS). Chromatography employed a reversed phase EASY-Spray^™^ column (25 cm × 75 μm ID, PepMap^™^ RSLC C18, 2 μm, ThermoFisher Scientific) connected to an UltiMate^™^ 3,000 RSLCnano HPLC (ThermoFisher Scientific). Mobile phase buffers A (0.1% formic acid) and B (0.1% formic acid in 100% acetonitrile) were delivered at 300 nl/min with the following gradient: 3–20% B in 115 min, 20–32% B in 19 min, 32–95% B in 1 min.

Data-dependent acquisition (DDA) mode was used to select ions for tandem MS. Positive ion precursor scans (375–1800 *m/z*) were acquired at 60,000 resolution with a normalized automatic gain control (AGC) target of 100%. Peptide ions were fragmented using higher-energy collisional dissociation (HCD) at a normalized collisional energy of 27%. Dynamic exclusion was applied for 45 s over ±10 ppm. MS/MS scans were collected with a first fixed mass of *m/z* 100, 2 *m/z* isolation window, 15,000 orbitrap resolution, and normalized AGC target of 100%.

### Hydrophilic interaction liquid chromatography

To expand the number of tryptic peptides detected and establish a list of target peptide ions for parallel reaction monitoring (PRM; Peterson et al., 2012) MS experiments, peptide products from eFASP were fractionated off-line by hydrophilic interaction chromatography (HILIC; Alpert, 1990). Tryptic peptides (50 μg) were loaded onto BioPureSPN MACRO PolyHYDROXYETHYL A^™^ columns (The Nest Group) in 90% acetonitrile and 150 mM ammonium formate, pH 3. Peptides were then eluted in six fractions sequentially using: 80, 78, 74, 71, 40, and 35.5% acetonitrile, respectively. Peptide eluates were dried, resuspended, and desalted with C18 STAGE tips as described above. For *S. wolfei* Göttingen only, fractions 1 and 6 were combined to reduce instrument operation time. *S. wolfei* subsp. *methylbutyratica* fractions 1 and 6 were analyzed independently to optimize detection of hydrophobic, long-chain acyl-PTMs present in fraction 1. HILIC fractionated peptides were analyzed by LC–MS/MS as described above.

### Acyl quantification

Unscheduled PRM-targeted MS analysis (Peterson et al., 2012) was utilized to quantify acyl-peptides of interest using the list of target peptide ions identified in HILIC DDA experiments. Desalted peptides from the eFASP procedure were analyzed without offline fractionation. Liquid chromatography was performed identically to the shotgun and HILIC-fractionated experiments. The inclusion list used for PRMs is shown in Supplementary Table 1. The MS2 scans were obtained at a 17,500 resolution with a normalized AGC target 100% and an isolation window of *m/z* 1.2 using HCD fragmentation at a normalized collision energy of 30%.

### Analysis of acyl-peptide standards to validate identifications

Synthetic acylated peptide standards, TPIGK (valeryl) FLGQFK and TPIGK (hexanyl) FLGQFK, (1 pmol each) were desalted with STAGE tips and delivered for tandem MS in DDA mode, using the same LC–MS/MS parameters as described above.

### Data analysis

RAW data files from DDA experiments were analyzed using ProteomeDiscoverer^™^ (version 1.4) and database searched with the Mascot search algorithm (Matrix Science, version 2.6.2; Perkins et al., 1999). UniProt *S. wolfei* Göttingen and *M. hungatei* protein sequences (as of February 1, 2017) were combined with sequences of common contaminants into a database totaling 5,691 entries and searched against samples from the respective cell cultures. *S. wolfei* subsp. *methylbutyratica* protein sequences from the draft genome available at JGI (Narihiro et al., 2016) and UniProt *M. hungatei* protein sequences (as of April 7, 2020) were similarly concatenated to contaminant sequences into a database totaling 6,124 entries. Parameters selected for the Mascot search were: enzyme name, trypsin; maximum missed cleavage sites, 2; precursor mass tolerance, 10 ppm; fragment mass tolerance, 0.02 Da; and variable methionine oxidation and cysteine carbamidomethylation. Searches also considered the potential acyl-lysine modifications listed in Supplementary Table 2. The selection of acyl modifications to be searched was based on RACS attributed to the β-oxidation pathways of *S. wolfei* Göttingen and *S. wolfei* subsp. *methylbutyratica*. A requirement for protein identification was that at least 2 unique peptides be detected, each with a Mascot ion score ≥ 20, which corresponds to a 95% confidence. Protein abundances were normalized by species to account for differences in cell pellet compositions between mono and cocultures. Proteomic datasets submitted to the ProteomeXchange Consortium through the MassIVE repository are identified as PXD034881.

For the targeted analyses (PRM experiments), ProteomeDiscoverer^™^ search results from HILIC fractions were imported into Skyline (version 21.1.0.278, MacCoss lab, University of Washington) to build spectral libraries (Maclean et al., 2010). PRM RAW files were imported into Skyline for quantitative data processing and proteomic analysis. The protein sequence database of *S. wolfei* Göttingen described above was added to Skyline as the background proteome. Peak areas from extracted ion chromatograms for four to six coeluting product ions were selected for quantification based on a higher dot product correlation between observed transitions of target peptides and library spectrum, indicating higher confidence in peptide detection. Cyclized immonium ions (Muroski et al., 2021) for acyl-lysine modifications were added to the Skyline analysis as customized product ions. All integrated peaks were manually inspected to ensure correct peak detection and integration. Peak areas of selected product ions were summed for acyl-peptide quantification. As an internal control, the abundance of each acyl-peptide was normalized to the abundance of an unmodified peptide in the same protein. Skyline files have been deposited to Panorama and can be accessed.^1^

### Protein expression and purification of *Syntrophomonas wolfei* Göttingen Act2 (Swol_0675)

The Swol_0675 gene was synthesized and cloned into pMAPLe4 (Arbing et al., 2013; Twist Bioscience) which appends an N-terminal tag encoding a hexahistidine tag and TEV protease cleavage site to the target protein. The expression plasmid was transformed into *E. coli* BL21-Gold (DE3) and an overnight inoculum was used to inoculate 2 l of Terrific broth supplemented with kanamycin. Cultures were grown at 37°C and protein expression was induced by the addition of IPTG to 0.5 mM when the culture reached an OD_600_ of 1.0; growth was continued at 18°C overnight and the cells harvested by centrifugation approximately 18 h after induction. The cell pellet was resuspended in Buffer A (50 mM Tris pH 8.0, 300 mM NaCl, 20 mM imidazole, 5 mM β-mercaptoethanol) supplemented with EDTA (1 mM), PMSF (1 mM), and benzonase. The cell suspension was lysed using an Emulsiflex C-3 and the lysate clarified by centrifugation (39kxG, 40 min). The clarified lysate was loaded on a 5 ml NiNTA column equilibrated in Buffer A and the column was washed extensively with buffer A before eluting bound protein with a linear gradient of Buffer B (Buffer A with 300 mM imidazole). TEV protease was added to the peak fractions and the digest was dialyzed against Buffer A overnight. The following day the digest was passed over the NiNTA column to remove H6-tagged TEV protease and the cleaved affinity tag and the flow-through were collected. The flow-through was concentrated and further purified by size exclusion chromatography using Superdex 200 equilibrated with 20 mM Tris pH 8.0, 150 mM NaCl, 5 mM β-mercaptoethanol. The peak containing Act2 was concentrated using an Amicon^®^ Ultra-15 centrifugal filter unit [10 kDa molecular weight cut-off (MWCO)] to 30 mg/ml.

### Crystallization and structure determination of *Syntrophomonas wolfei* Göttingen Act2 (Swol_0675)

Crystals of Act2 were grown at room temperature using the hanging drop vapor diffusion method with 0.1 M sodium formate pH 7.0, 12% polyethylene glycol (PEG) 3,350 as the reservoir solution. Crystals were cryoprotected with a brief soak in 23% PEG 3350, 5% PEG 400 before being flash frozen. Diffraction data was collected at the Northeastern Collaborative Access Team (NE-CAT) facility, beamline 24-ID-C, at the Advanced Photon Source at Argonne National Laboratory. Data were processed with XDS (Kabsch and IUCr, 2010) and the structure solved by molecular replacement using the program phenix.phaser (McCoy et al., 2007) with a search model generated by the Phyre2 server (Kelley et al., 2015). The model was refined with phenix.refine (Afonine et al., 2012). Crystallographic data and refinement statistics are listed in Table 1 (prepared with phenix.table_one) and the structure has been deposited in the Protein Data Bank under accession code 7N7Z.

**Table 1:** Data collection and structural refinement statistics of *Syntrophomonas wolfei* Göttingen Act2.

*Syntrophomonas wolfei* Göttingen Act2 (Swol_0675) (PDB entry: 7N7Z)	
Wavelength	0.9792
Resolution range	41.64–2.022 (2.094–2.022)
Space group	P 41 21 2
Unit cell	81.07 81.07242.31 90 90 90
Total reflections	317,087 (26164)
Unique reflections	53,598 (5118)
Multiplicity	5.9 (5.1)
Completeness (%)	99.40 (96.93)
Mean I/sigma(I)	10.47 (1.63)
Wilson B-factor	33
R-merge	0.1041 (0.8022)
R-meas	0.1141 (0.8933)
R-pim	0.04584 (0.3867)
CC1/2	0.997 (0.511)
CC*	0.999 (0.822)
Reflections used in refinement	53,598 (5118)
Reflections used for R-free	5,360 (512)
R-work	0.2327 (0.3383)
R-free	0.2743 (0.4035)
CC(work)	0.941 (0.670)
CC(free)	0.950 (0.445)
Number of non-hydrogen atoms	5,987
Macromolecules	5,768
Ligands	0
Solvent	219
Protein residues	795
RMS(bonds)	0.002
RMS(angles)	0.48
Ramachandran favored (%)	95.82
Ramachandran allowed (%)	3.93
Ramachandran outliers (%)	0.25
Rotamer outliers (%)	0
Clashscore	4.31
Average B-factor	42.66
Macromolecules	42.71
Solvent	41.43
Number of TLS groups	1

### *In vitro* acylation assay

Purified *S. wolfei* Göttingen Swol_0675 recombinant protein (Act2, 5 μM) was incubated either with 1 μM acetyl-CoA or 50 μM butyryl-CoA in 100 mM ABC for 1 or 3 h(s), respectively, at 37°C as described previously (Parks and Escalante-Semerena, 2020). Reactions were stopped by exchanging into 100 mM ABC buffer to remove excess acetyl-CoA using 10 kDa MWCO Amicon^®^ centrifugal filters (Millipore). Modified proteins were then digested for analysis by LC–MS/MS. Briefly, the modified proteins were heated to 95°C for 10 min, disulfides were reduced with 20 mM dithiothreitol (DTT) for 1 h at 60°C, and alkylated by incubation with 50 mM iodoacetamide at room temperature in the dark for 45 min. Following incubation, iodoacetamide was quenched by adding excess DTT. Acylated Swol_0675 was digested overnight with trypsin (1:100, 37°C), followed by a second overnight digest with endoproteinase GluC (1:100, 25°C). Peptides were desalted with STAGE tips. The mass spectrometer was operated in DDA mode, as described earlier, with the LC gradient: 3–20% B in 32 min, 20–32% B in 5 min, 32–95% B in 1 min. MS data was analyzed as described above.

## Results

### Comparing the proteomes of butyrate and crotonate grown *Syntrophomonas wolfei* Göttingen

**Figure 1:**
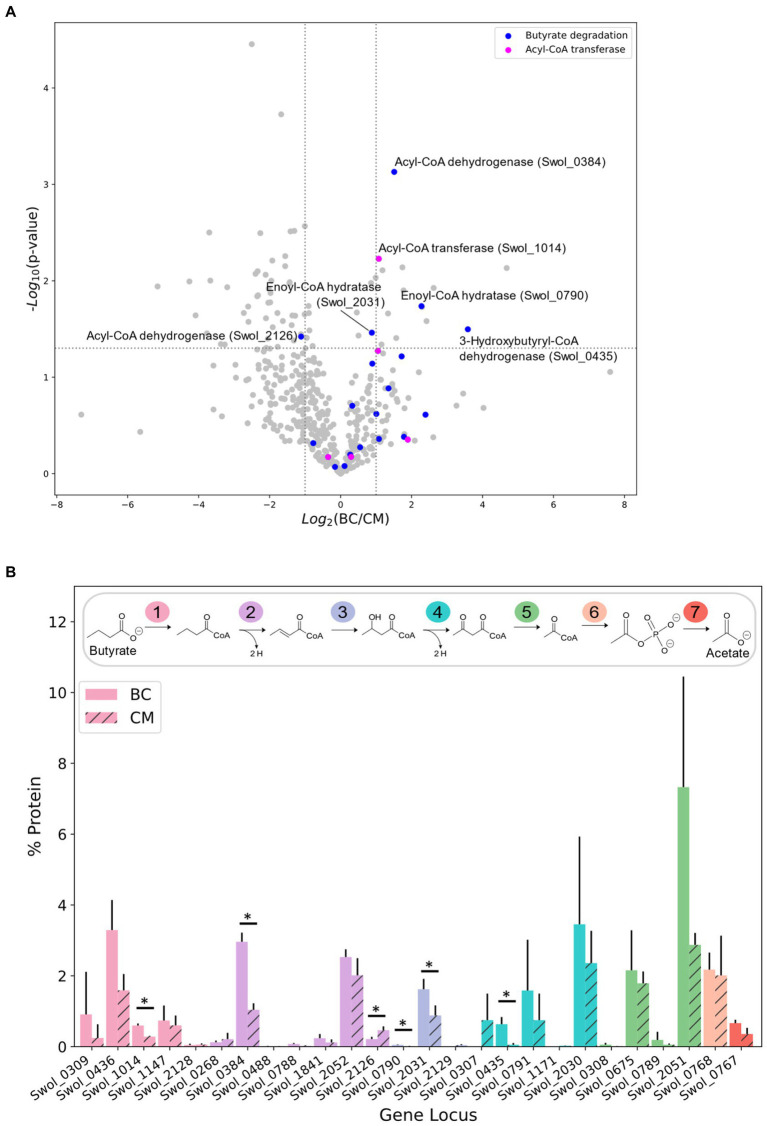
Protein abundance of *S. wolfei* Göttingen proteome between carbon substrates. **(A)** Volcano plot of protein-fold changes between butyrate cocultures (BC) and crotonate monocultures (CM). Dotted lines represent significance cutoffs (*p* < 0.05, fold-change >2 or < 0.5). **(B)** Percent protein abundance of beta-oxidation paralogs. Colors indicate the step catalyzed by the enzymes. Each number in pathway represents the step catalyzed by the paralogs. (*p < 0.05).

*Syntrophomonas wolfei* Göttingen metabolizes butyrate as shown in Figure 1B, relying on methanogen *M. hungatei* to maintain sufficiently low H_2_ and/or formate levels such that butyrate degradation is thermodynamically favorable. *S. wolfei* does not require a partner organism to metabolize crotonate, because for every molecule of crotonate oxidized down the pathway, another is reduced to butyrate to consume the electrons generated by crotonate oxidation. Whole cell extracts from *S. wolfei* Göttingen grown on crotonate in pure culture or on butyrate in coculture with *M. hungatei* were trypsin-digested and analyzed by tandem mass spectrometry to examine how carbon substrates influenced protein abundances. Overall abundance differences between the two carbon substrates are illustrated in Figure 1A. Out of the 446 proteins identified in *both* the crotonate monoculture (CM) and butyrate coculture (BC), only 71 proteins had significant changes in protein abundance (>2 × fold change, *p* < 0.05), with 57 and 14 proteins up-regulated in CM and BC cultures, respectively. Out of these 71, 39 proteins had abundance changes greater than 3-fold, and many of these changes were relatively modest, mirroring the observations from previous proteomic studies (Schmidt et al., 2013; Sieber et al., 2015; Crable et al., 2016).

Our MS analysis was able to achieve greater coverage of the proteome, identifying proteins not previously detected. Genomic and proteomic analysis has shown that *S. wolfei* Göttingen contains and simultaneously expresses multiple paralogs for each step in β-oxidation (Sieber et al., 2010, 2015). Improved MS technologies enabled our proteomic analyses to detect 15 additional low-abundance paralogs that were not seen in previous work (Figure 1B; Schmidt et al., 2013; Sieber et al., 2015; Crable et al., 2016). These identified proteins catalyze the first five steps in β-oxidation and are as follows: acyl-CoA transferase (Swol_0309, 1014, 1147, 2128), acyl-CoA dehydrogenase (Swol_0488, 0788, 1841), enoyl-CoA hydratase (Swol_0790, 2031, 2129), *3-*hydroxybutyryl-CoA dehydrogenase (Swol_0307, 0791, 1171), and acetyl-CoA acetyltransferase (Swol_0308, 0789). Our improved proteomic depth revealed additional β-oxidation paralogs and led us to investigate the presence of protein acyl-modifications which have been largely unexplored in *S. wolfei*.

### The *Syntrophomonas wolfei* Göttingen proteome has a wide range of acyl-lysine modifications

A study in another syntroph, *S. aciditrophicus*, identified several types of acylations related to the RACS generated in the microbe’s metabolism (Muroski et al., 2022). This investigation raised the possibility that acylations could be similarly abundant in other syntrophs, like *S. wolfei* Göttingen, which also produces RACS. To comprehensively identify acyl-lysine modifications in *S. wolfei*, we performed shotgun proteomics *without utilizing PTM-specific enrichment*. Protein lysates were also pre-fractionated by hydrophilic interaction chromatography (HILIC) to achieve greater depth in analyzing the acylome. Database searches incorporated mass shifts corresponding to putative modifications arising from RACS formed during butyrate and crotonate degradation: acetyl-, butyryl-, crotonyl, *3-*hydroxybutyryl, and acetoacetyl-lysine (Supplementary Table S2). Of the 5 types of acylation predicted, we identified 4 (acetyl-, butyryl-, crotonyl-, and *3-*hydroxybutyryl-lysine) across both culture conditions. A total of 238 sites were identified in 111 proteins in BC grown cells and a total of 226 sites in 176 proteins in CM grown cells. These are large numbers for un-enriched samples. For comparison, enrichment for acetylated peptides from MV4-11 cells, a human acute myeloid leukemia cell line, using an antibody against acetyl-lysine identified 3,600 acetylation sites on 1750 proteins, and experiments without antibody enrichment resulted in a 60-fold decrease in acetylation sites (Choudhary et al., 2009).

**Figure 2:**
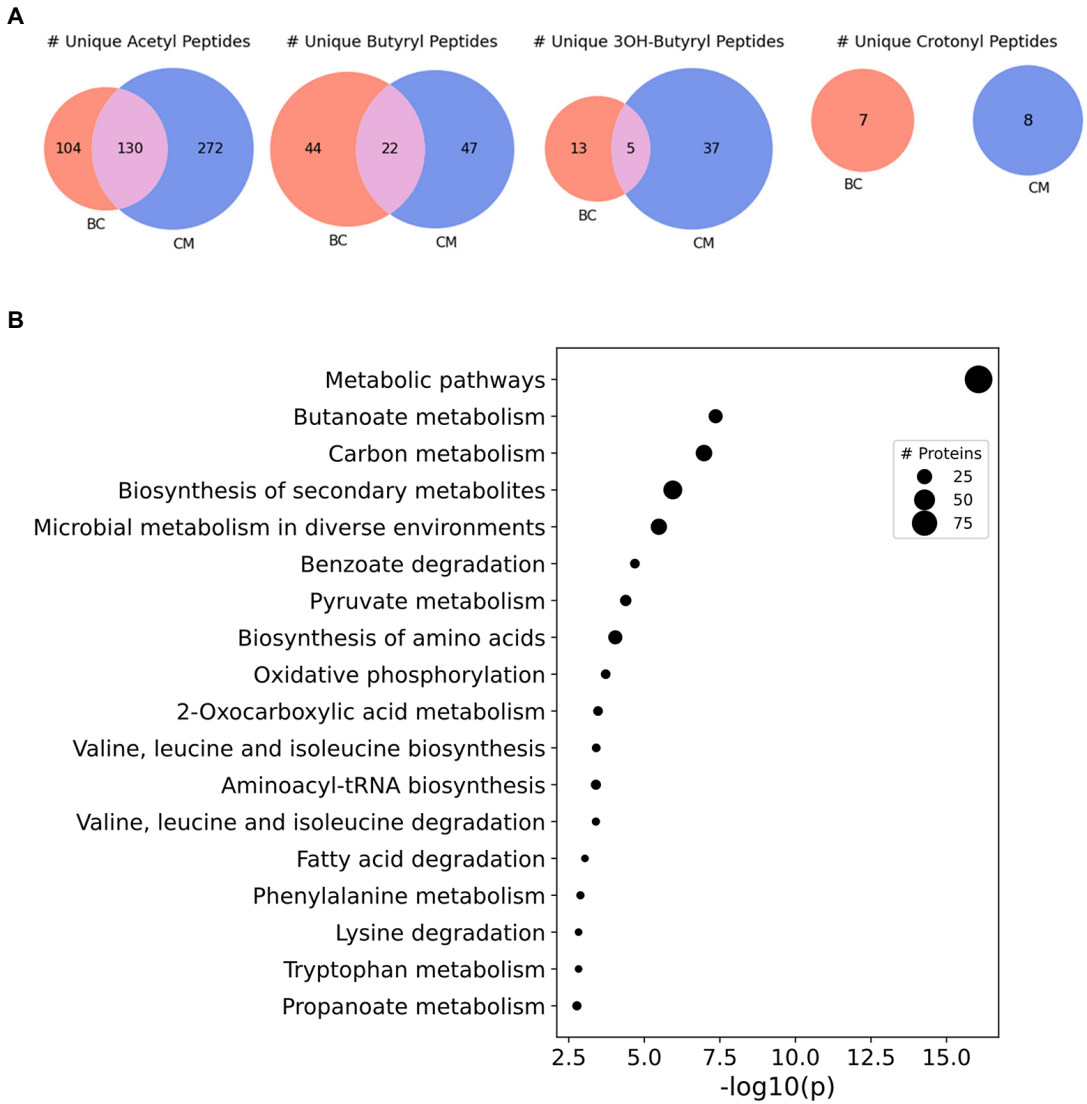
Acyl-lysine modifications identified across culture conditions in *S. wolfei* Göttingen. **(A)** Unique acyl-peptides identified between growth conditions BC and CM. **(B)** Significantly enriched pathways (KEGG Ontology) for the acylated proteins identified across both culture conditions. Point size illustrates the number of proteins identified.

Of the acyl-PTMs identified in *S. wolfei* Göttingen, acetylation was most common, followed by butyrylation, *3-*hydroyxbutyrylation, and crotonylation, respectively (Table 2). Some lysine residues were identified with a variety of acyl-modifications; e.g., K159 from acyl-CoA transferase (Swol_1014) was observed with either an acetyl- or butyryl-lysine modification (Table 3). These PTMs each count towards the number of sites identified for their respective modification, but the lysine residue is only counted once in the total number of acyl-lysine sites identified. The type of acylation observed varies with culture condition, as displayed in Figure 2A. The number of unique acyl-peptides, a metric commonly reported in MS proteomics, encompasses peptides that contain multiple modified lysines, providing additional context that would be otherwise missed from reporting individual modified sites. Supplementary Table 3 lists the modified peptide sequences detected. An important caveat is that the modification differences with carbon substrate may simply reflect interference from the *M. hungatei* proteins present in butyrate cocultures, reducing proteomic coverage of *S. wolfei*. To gauge this effect, we compared the number of *S. wolfei* proteins identified from the HILIC-fractionated butyrate cultures to those from crotonate. While 12% more *S. wolfei* proteins were identified from crotonate monocultures (981 vs. 873), a majority (768) of proteins was identified from both conditions, suggesting that the observed acylation differences primarily reflect the carbon substrate (Supplementary Figure 1). Notably, all types of acyl-PTMs are present in both conditions, as is evident, for example, from butyryl-lysine being identified in crotonate cultures. Butyryl-CoA, the respective RACS for butyrylation, is known to form in crotonate grown cells from the reduction of crotonyl-CoA during crotonate fermentation (McInerney et al., 2009). Observation of butyryl modification is also consistent with the fact that certain enzymatic steps in β-oxidation are reversible in syntrophs (James et al., 2019). Indeed, the capability of *S. wolfei* to dismutate crotonate to acetate and butyrate is key to its axenic cultivation (McInerney and Beaty, 1988).

**Table 2:** Overall acylation sites detected in *S. wolfei* Göttingen.

	Butyrate coculture		Crotonate monoculture	
Modification Type	Sites identified	Proteins identified	Sites identified	Proteins identified
Acetyl	213	86	365	167
Butyryl	62	38	50	27
*3-*hydroxybutyryl	16	15	35	29
Crotonyl	7	7	8	7

See materials and methods.

**Table 3:** Acylation sites in β-oxidation pathway enzymes of *S. wolfei* Göttingen.

Step	Protein accession	Position in protein	Modification type(s) and position(s) in peptide	Sequence
1	Swol_0436	10	K1(Acetyl)	KLVSADEAVK
1	Swol_0436	211	M9(Oxidation); K10(Acetyl)	AAELIIEEMKDECCVQLGIGGMPNAVGK
1	Swol_0436	375	K2(Acetyl, Butyryl, *3-*Hydroxybutyryl)	SKIVPTLKPGAIVTDPR
1	Swol_0436	381	K6(Acetyl, Butyryl, Crotonyl)	IVPTLKPGAIVTDPR
1	Swol_0436	373, 375	K2(Acetyl); K4(*3-*Hydroxybutyryl)	LKSKIVPTLKPGAIVTDPR
1	Swol_0436	373, 375	K2(Acetyl); K4(Acetyl)	LKSKIVPTLKPGAIVTDPR
1	Swol_0436	375, 381	K2(Acetyl); K8(Acetyl)	SKIVPTLKPGAIVTDPR
1	Swol_0436	375, 381	K2(Butyryl); K8(Acetyl)	SKIVPTLKPGAIVTDPR
1	Swol_0436	375, 381	K2(*3-*Hydroxybutyryl); K8(Acetyl)	SKIVPTLKPGAIVTDPR
1	Swol_1014	41	K4 (Acetyl)	TLDKALAK
1	Swol_1014	47	K1(Acetyl)	KDELSDVK
1	Swol_1014	151	K3 (Acetyl)	AQKIIVEVNEK
1	Swol_1014	159	K8 (Acetyl, Butyryl)	IIVEVNEKQPR
1	Swol_1014	374	K7(Acetyl)	AADGSIKSR
1	Swol_1014	432	K5(Acetyl)	DELIKAAQEQGIWR
1	Swol_1147	10	K7 (Acetyl, *3-*Hydroxybutyryl)	TYLDEYKEK
1	Swol_1147	21	K7 (Acetyl)	TADEAVKVVK
1	Swol_1147	434	K18 (Acetyl, Butyryl)	AEALINIAHPDLRDELVKEAQK
1	Swol_1932	4	K3(Acetyl)	YQKLLEEYK
1	Swol_1932	12	K2(Butyryl)	SKLVTADEAAK
2	Swol_0268	179	K5(Acetyl); M7(Oxidation)	SLGGKGMSAFIISK
2	Swol_0268	194	K6(Acetyl)	DNPGLKVGQHFYK
2	Swol_0268	227	K6(Acetyl)	EDLLGKEGQGLQIAMSSFDHGR
2	Swol_0268	273	K5(Acetyl)	VQFGKPISK
2	Swol_0384	42	K4(Acetyl, Butyryl)	DDIKPVLGPILK
2	Swol_0384	343	K6(Acetyl, Butyryl, *3-*Hydroxybutyryl)	LLTNPKAGR
2	Swol_0384	393	K1(Acetyl); C14(Carbamidomethyl)	KAASDLIEITTPLCK
2	Swol_0384	495	K7(Acetyl)	GNAALSKEFAILDK
2	Swol_0384	502	K7(Acetyl)	EFAILDKALK
2	Swol_0384	505	K3(Acetyl)	ALKAYQEMLK
2	Swol_0384	512	M5(Oxidation); K7(Acetyl)	AYQEMLKVYAGYAK
2	Swol_0384	554	K11(Acetyl, Butyryl)	QILDQAVLADKK
2	Swol_1841	18	K1(Acetyl)	KFSENEIAPLVK
2	Swol_1841	310	K5(Acetyl)	ASEGKELASDAAR
2	Swol_2052	14	K3(Acetyl)	DIKFQIK
2	Swol_2052	69	M7(Oxidation); K8(Acetyl, Butyryl, *3-*Hydroxybutyryl)	ESDEIGMKHVGGNEK
2	Swol_2052	76	K7(Acetyl, Butyryl)	HVGGNEKAVISPDVFK
2	Swol_2052	226	K5(Acetyl); C13(Carbamidomethyl)	DPDAKPGTAGISCLVVPK
2	Swol_2052	344	K5(Acetyl, Butyryl, *3-*Hydroxybutyryl)	STDPKGPSVR
2	Swol_2052	475	K6(Acetyl)	KGEPFKK
2	Swol_2052	476	K1(Acetyl)	KWLAEIGDFIANKK
2	Swol_2052	489	K1(Acetyl); M11(Oxidation); M12(Oxidation)	KTPEFAAEFAMMEK
2	Swol_2052	522	M11(Oxidation); K20(Acetyl)	AFAAFNSIIDMNAAWTTTNKQLK
2	Swol_2052	525	K3(Butyryl)	QLKQLFATR
2	Swol_2052	570	K15(Acetyl)	LAELGDSHFDANFYKGK
2	Swol_2126	22	K5(Acetyl)	EFAEKSIAPVAK
2	Swol_2126	117	K2(Acetyl)	AKHIVSFAFTEPGTGSDPK
2	Swol_2126	214	K3(Acetyl)	DVKVPADNLLGK
2	Swol_2126	273	K6(Acetyl)	GQPIAKFQAIQLK
3	Swol_2031	197	K18(Acetyl)	IGLVNHVYPADQLMDEAKK
3	Swol_2031	202	K4(Acetyl, *3-*Hydroxybutyryl)	IANKAPLAVGYAK
3	Swol_2,129	15	K3(Acetyl)	LEKGILLVSLNRPEK
4	Swol_0435	52, 56	K1(Acetyl); K5(Butyryl)	KAVEKGK
4	Swol_0435	172	K23(Acetyl)	LVEVIPGAETSEAVSSAIVELCKK
4	Swol_0791	28	K1(Acetyl)	KVVLYDIK
4	Swol_0791	35	K7(Acetyl)	VVLYDIKQEFVDR
4	Swol_0791	48	K7(Acetyl)	AIAAIGKSLAK
4	Swol_0791	52	K3(*3-*Hydroxybutyryl)	SLAKAEER
4	Swol_0791	182	K2(Acetyl)	VKEGPGFVVNR
4	Swol_2030	37	M9(Oxidation); K10(Acetyl, Butyryl)	NVILYDIDMKFVDK
4	Swol_2030	41	K4(Acetyl, Butyryl, Crotonyl)	FVDKAIGAIK
4	Swol_2030	47	K6(Butyryl)	AIGAIKK
4	Swol_2030	52	K4(Butyryl)	GLTKAEEK
4	Swol_2030	56	K4(Crotonyl)	AEEKGKAAPGTADAVVGR
4	Swol_2030	58	K2(Acetyl, Butyryl, Crotonyl, *3-*Hydroxybutyryl)	GKAAPGTADAVVGR
4	Swol_2030	131	M4(Oxidation); M5(Oxidation); K28(Acetyl)	LDAMMGPDVILATNTSSLSITEIAAITKR
4	Swol_2030	180	K8(Acetyl)	EIGKTPVKVK
4	Swol_2030	182	K2(Acetyl)	VKEGPGFVVNR
4	Swol_2030	263	K8(Acetyl, *3-*Hydroxybutyryl)	YRPAPPLKQLVR
4	Swol_2030	277	K3(Acetyl)	TGKGFYDYK
5	Swol_0308	210	K1(Butyryl)	KGITIIETDEHPIR
5	Swol_0675	75	M4(Oxidation); K5(Acetyl, Butyryl, Crotonyl, *3-*Hydroxybutyryl)	IIGMKVGLPVR
5	Swol_0675	191	C3(Carbamidomethyl); K16(Acetyl)	EECDALALTSHQNAVKAVDEGIFDR
5	Swol_0675	208	K8(Acetyl, Butyryl)	EIVPVVIKSK
5	Swol_0675	214	K3(Acetyl)	GDKVISKDEHPIR
5	Swol_0675	218	K4(Acetyl)	VISKDEHPIR
5	Swol_0675	233	M7(Oxidation); K9(Acetyl, Butyryl)	GASLETMAKLPPAFK
5	Swol_0675	304	K3(Acetyl)	VLKQAGWK
5	Swol_0675	381	K1(Acetyl); C16(Carbamidomethyl); M23(Oxidation)	KGGVVTAANASGINDCAAAAVFMSK
5	Swol_0789	335	M1(Oxidation); K3(Acetyl); M10(Oxidation)	MLKEDFGIEMDLSK
5	Swol_1934	121	K17(Acetyl)	AGDADIIMAGGTENMDKAPFILPNAR
5	Swol_1934	210	K2(Acetyl); K3(Butyryl)	GKKGDIVFDTDEHPR
5	Swol_1934/2051	45	K4(Acetyl); C15(Carbamidomethyl)	AGIKAEQIDEVIFGCVLQAGLGQNVAR
5	Swol_1934/2051	199	K2(Acetyl, Butyryl, *3-*Hydroxybutyryl)	FKDEIVPVVIK
5	Swol_1934/2051	224	K1(Acetyl, Butyryl); K7(Oxdiation)	KSTPEAMAK
5	Swol_1934/2051	232	M6(Oxidation); K8(Acetyl, Butyryl)	STPEAMAKLAPAFK
5	Swol_1934/2051	265	K9(Acetyl); M11(Oxidation)	EKADELGIKPMAK
5	Swol_2051	23	K11(Acetyl, Butyryl); M23(Oxidation)	TPVGTFGGTIKDVGAADLGALVMGEAITR
5	Swol_2051	78	K5(Acetyl, Butyryl)	AGIPKEITAFTINK
5	Swol_2051	104	K10(Acetyl)	AVSLAAQVIKAGDADIILAGGTENMDK
5	Swol_2051	121	K17(Crotonyl)	AGDADIILAGGTENMDKAPFLLPNAR
5	Swol_2051	191	K7(Acetyl, Butyryl, *3-*Hydroxybutyryl)	SQDLAAKAIESGR
5	Swol_2051	211	K1(Acetyl, Butyryl, Crotonyl, *3-*Hydroxybutyryl)	KGDTVFDTDEHPR
5	Swol_2051	238	K6(Acetyl, Butyryl)	LAPAFKK
5	Swol_2051	239	K1(Acetyl, Butyryl); M23(Oxidation)	KGGSVTAGNASGINDGAAAVIVMSK
5	Swol_2051	263	M23(Oxidation); K25(Acetyl)	KGGSVTAGNASGINDGAAAVIVMSKEK
6	Swol_0768	2	M1(Oxidation); K2(Acetyl)	MKVLVVNAGSSSIK
6	Swol_0768	110	K19(Acetyl)	VVHGGEGFAESVVIDDEVKR
6	Swol_0768	270	M3(Oxidation); K14(Acetyl, Butyryl)	LGMNSSEANNYFNKK
6	Swol_0768	271	K1(Acetyl); M4(Oxidation)	KSGMLGLSGVSNDLR
6	Swol_0768	364	K2(Acetyl, Butyryl, *3-*Hydroxybutyryl)	GKEVDVATADSK
6	Swol_0768	374	K10(Acetyl)	EVDVATADSKVR
7	Swol_0767	53	K14(Acetyl)	VARPILLGDEGAIKGIASK
7	Swol_0767	233	M1(Oxidation); K9(Acetyl)	MVQATAIAKEK
7	Swol_0767	292	K3(Acetyl)	TAKAEAIGPILQGIAKPVNDLSR

Gene/protein locus tag destinations from the IMG JGI genome database.

### Enzymes involved in *Syntrophomonas wolfei* Göttingen β-oxidation are extensively acylated

**Figure 3:**
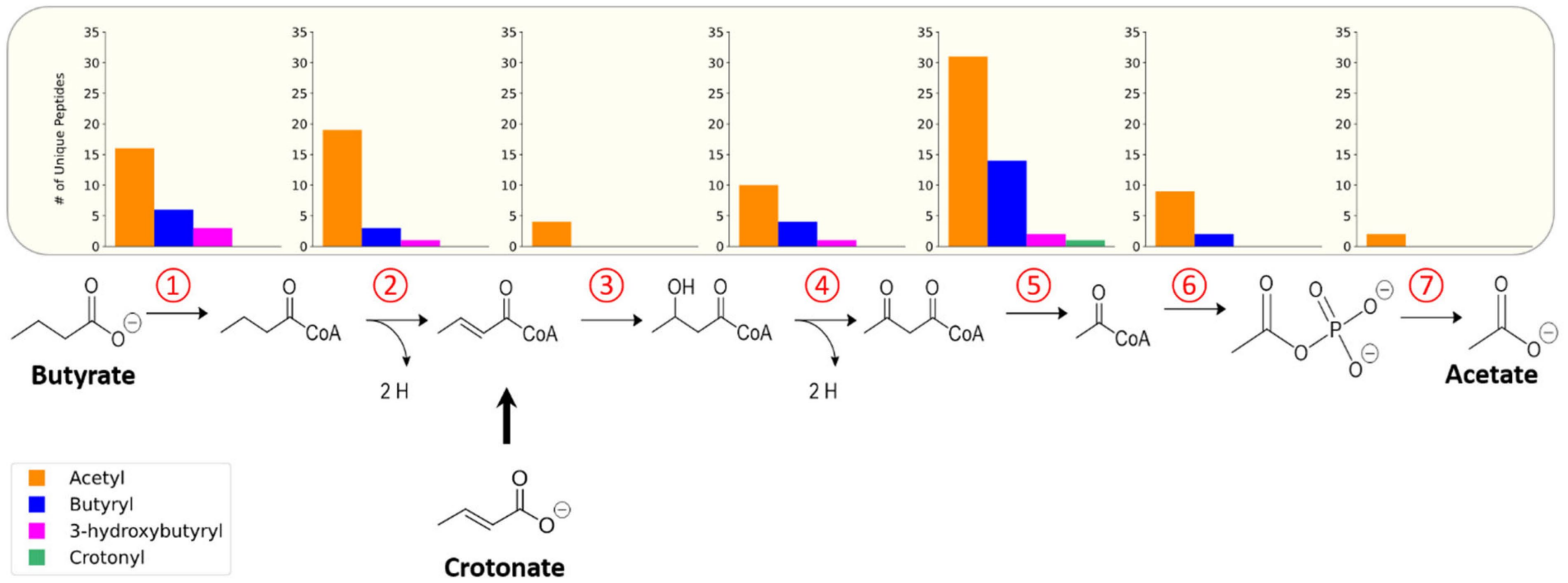
Acylated proteins identified in butyrate degradation pathway of *S. wolfei* Göttingen. Number of unique acylated peptides identified for each step in the pathway. Each number represents the step in the pathway catalyzed by the paralogs.

To understand the functional impact of these acyl-PTMs, a KEGG (Kyoto Encyclopedia of Genes and Genomes) pathway enrichment analysis was performed on the modified proteins (Kanehisa and Goto, 2000; Kanehisa and Sato, 2020). The most enriched processes were involved in metabolism, including the general categories butanoate metabolism, carbon metabolism, biosynthesis of secondary metabolites, and microbial metabolism in diverse environments (Figure 2B). Indeed, enzymes involved in steps 1,2,4, and 5 of the butyrate β-oxidation pathway were heavily acylated, with 175 unique modified peptides identified on 21 out of the 26 proteins (Table 3). In comparison, enzymes catalyzing glycolysis and gluconeogenesis had fewer sites of acylation; 9 unique modified peptides were identified on 5 out of the 26 proteins (Supplementary Table 3). As there seemed to be a relationship between metabolic pathways producing RACS and protein acylation, we focused on the butyrate β-oxidation pathway. β-oxidation paralogs were grouped by the reaction catalyzed to discern trends in the number and type of acyl modifications identified. Figure 3 shows that populations of enzymes involved in all of the catalytic steps are modified, with the largest number of unique, modified peptides found for acetyl-CoA transferase and the smallest number found for enoyl-CoA hydratases and acetate kinase. For the enzymes involved in β-oxidation, acetylation is the primary acylation for each of the steps in the pathway, followed by butyryl-, *3-*hydroxybutyryl-, and crotonyl-lysine modification. The low number of crotonyl-lysine modified peptides detected could be due to the very high activity of enoyl-CoA hydrolase in *S. wolfei* (McInerney and Wofford, 1992). This enzyme, which catalyzes the third step in β-oxidation, converts crotonyl-CoA to *3-*hydroxybutyryl-CoA and its high activity generates a smaller pool of crotonyl-CoA as compared to that of other RACS. The relative abundance of each type of acylation may be related to the intracellular concentrations of the respective acyl-CoA. The relative abundance of each acyl modification for enzymes involved in β-oxidation is similar to the acylation trends observed among all acylated proteins (those involved in other biological processes besides β-oxidation) in the *S. wolfei* proteome.

Many of the proteins involved in butyrate degradation that were similarly abundant in BC and CM grown cultures were found modified by several acyl-PTMs. Although proteins with significant abundance changes were also observed to be modified, these constitutively expressed proteins, Swol_0436, Swol_2052, Swol_2030, and Swol_2051 from steps 1,2,4, and 5, respectively, are of special interest. These proteins (Table 3) were particularly abundant compared to paralogs of the respective enzyme, maintained relatively stable expression (abundance) levels across growth conditions, and exhibited acylated residues. The presence of modifications on these proteins could suggest that post-translational regulation was in play.

### Quantitation of *Syntrophomonas wolfei* Göttingen acyl-peptide abundances between cell growth conditions

In addition to investigating the presence, type, and number of modified sites, we next sought to measure the relative abundance of these acyl-lysine modifications in butyrate- and crotonate-grown cells. Targeted MS experiments, known as parallel reaction monitoring (PRM), were performed on the acyl-lysine peptides listed in Table 3. In PRM, the mass spectrometer detects ions for a user-selected peptide at a defined mass-to-charge ratio, often within a specified time interval, and the instrument isolates this precursor ion for fragmentation. This process enables acquisition of tandem mass spectra across the peptide’s elution period, yielding fragment ion chromatograms from which abundance is determined, allowing for a highly specific and selective MS approach for targeted peptide quantitation (Peterson et al., 2012). One of the key challenges in quantitatively comparing proteins from microbial consortia is that the relative distribution of organisms comprising the population (e.g., co- versus mono-culture) influences peptide abundance measurements (Chignell et al., 2018). To overcome this limitation, peptides that were only observed unmodified from the respective acylated proteins were used as internal standards to normalize acylated peptide levels. Due to some modified peptides appearing in only one of the two culture conditions and peptide ionization efficiency limitations, not all acyl-peptides could be quantified. The selected ion library employed to quantify peptides of interest is shown in Supplementary Table 1. Targeted PRM capability is critical to quantify and localize PTMs, especially in peptides containing multiple modifications and for peptides that can be modified by different acyl groups. MS/MS level quantitation from PRM provides information on individual product ions, allowing confident discrimination between contributions from acyl-peptide variants. The utility of incorporating immonium ion derivatives for improved quantitation of acyl-peptides in PRM analyses is illustrated in Supplementary Figure 2 (Muroski et al., 2021).

**Figure 4:**
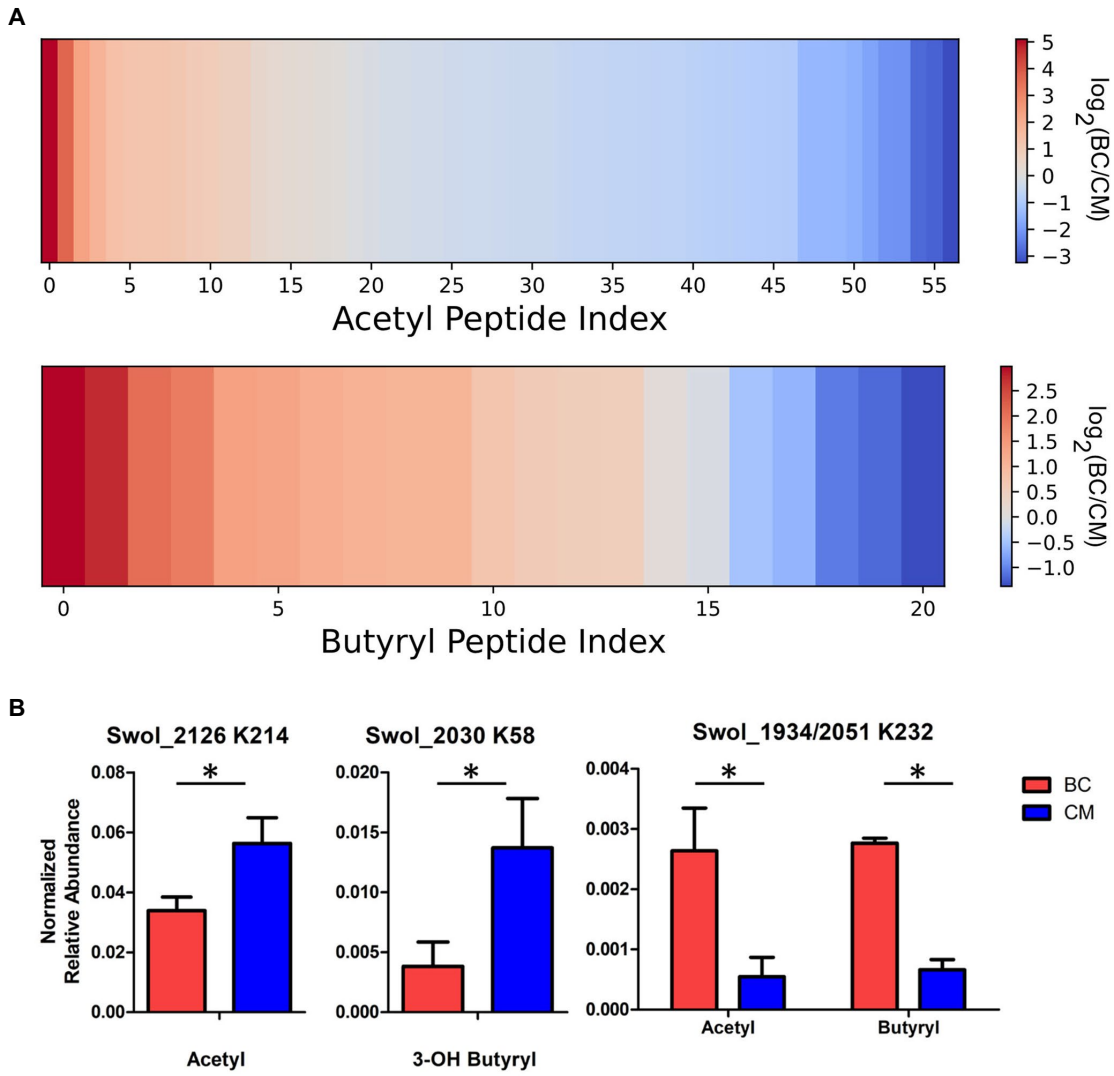
Acyl-peptide abundances vary between carbon substrates. **(A)** Heat map illustrating the fold change between growth conditions for acetyl- and butyryl-lysine peptides involved in the butyrate degradation pathway of *S. wolfei* Göttingen. **(B)** Acyl-peptide abundances in BC and CM cultures (*p < 0.05).

Acyl-peptide abundances differ in butyrate and crotonate grown cells. The data is complex, but trends can be identified. A majority of acetylated peptides were more abundant in crotonate cultures, while the majority of butyrylated peptides increased in butyrate cultures (Figure 4A). Some β-oxidation proteins changed abundance significantly, both at the protein level and at modified residues. One such case is acyl-CoA dehydrogenase (Swol_2126), which was observed in the shotgun proteomic measurements to increase in crotonate cultures; targeted PRM analyses revealed that K214 acetylation also increased significantly in crotonate grown cells (Figure 4B). Some of the constitutively expressed proteins noted earlier were also found to significantly vary in acylation levels. *3-*hydroxybutyryl CoA dehydrogenase (Swol_2030) had displayed increased *3-*hydroxybutyrylation on K58 in CM cultures. K8 of peptide STPEAMAKLAPAFK, common to K232 of acetyl-CoA transferases Swol_2051 and Swol_1934 (93% identical), showed increased acetylation and butyrylation when grown in BC (Figure 4B). The increased acetylation of K232 from butyrate grown cells deviates from the trend of acetylation levels generally increasing in crotonate-grown cells, highlighting the complexity of these modifications. That some acylation levels change significantly with cultivation condition, while others are stable, could suggest that regulatory mechanisms for lysine acylation are operating in *S. wolfei.*

### Uncovering acyl-PTM crosstalk in *Syntrophomonas wolfei* Göttingen

**Figure 5:**
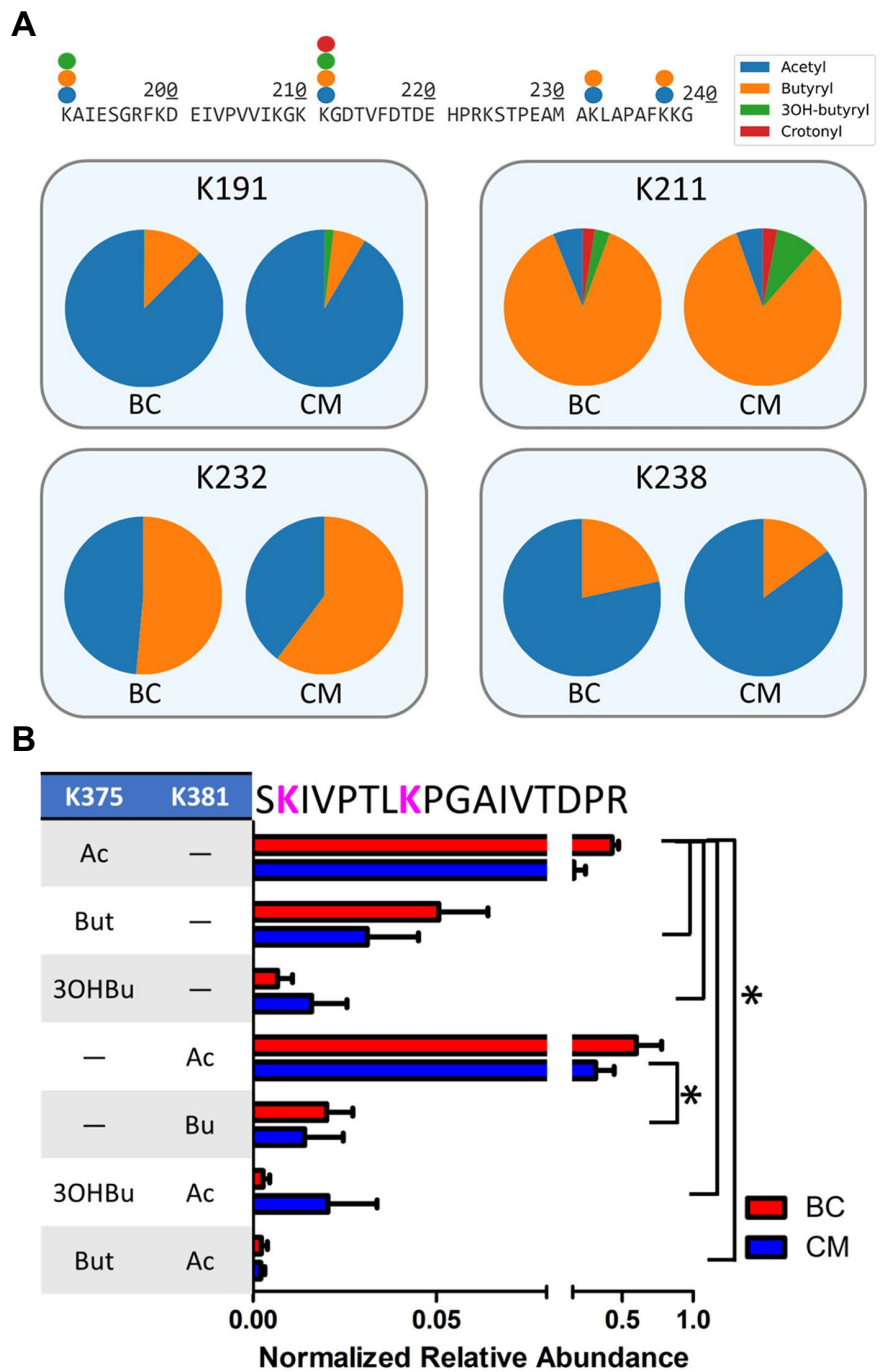
Crosstalk between acylation sites in β-oxidation enzymes. **(A)** Partial sequence (residues 190–240) of *S. wolfei* Göttingen acetyl-CoA transferase (Swol_2051) indicating sites and types of acyl-modifications. Pie charts depict relative site occupancy. **(B)** Relative abundance of different acyl combinations on peptide SKIVPTLKPGAIVTDPR (Swol_0436, residue 374–390; *p < 0.05).

Bypassing PTM enrichment not only provides a comprehensive view of acyl-modifications proteome-wide, but also allows for the detection of peptides bearing mixed acylations and for identifying PTM cross talk, which is the combination of different PTMs that can alter protein activity (Venne et al., 2014; Leutert et al., 2021). Many examples of intra-protein PTM crosstalk were observed, including instances of different acyl-PTMs modifying the same residue and peptides harboring multiple acyl-PTMs. In the β-oxidation pathway, 30 sites on 11 proteins were found where acyl-groups competed to modify the same residue (Table 3). Enzymes with similar abundances between BC and CM cultures (Swol_0436, Swol_2052, Swol_2030, and Swol_2051) were all found with lysine residues modified by a variety of acyl-groups. The relative proportion of each type of acyl-PTM shifted between BC and CM grown cells, as illustrated by the acyl-lysine site occupancies of K78, K191, K211, K232, and K238 of acetyl-CoA transferase Swol_2051 (Figure 5A). These data highlight that the type and abundance of acyl-PTMs are dynamic and fluctuate with changing metabolism.

Peptides containing multiple acyl modifications were also identified, potentially supporting crosstalk between proximal sites. Peptide SKIVPTLKPGAIVTDPR from acyl-CoA transferase (Swol_0436, residue 374–390) was found with 7 different acyl-PTM combinations at K375 and K381, with some versions either singly- or doubly- modified by acetyl, butyryl, or *3-*hydroxybutyryl-lysine(s). The singly-acetylated peptides, either at K375 or K381, were the most abundant out of the PTM combinations (Figure 5B). Not every acylation combination was identified on K375 and K381, suggesting that there may be some regulation of these PTMs. Proteins involved in *S. wolfei* β-oxidation are generally not modified by single, isolated groups, but rather by diverse combinations that could tune enzymatic function.

### Novel acyl-lysine modifications in *Syntrophomonas wolfei* subsp. *methylbutyratica* are metabolite-derived

**Figure 6:**
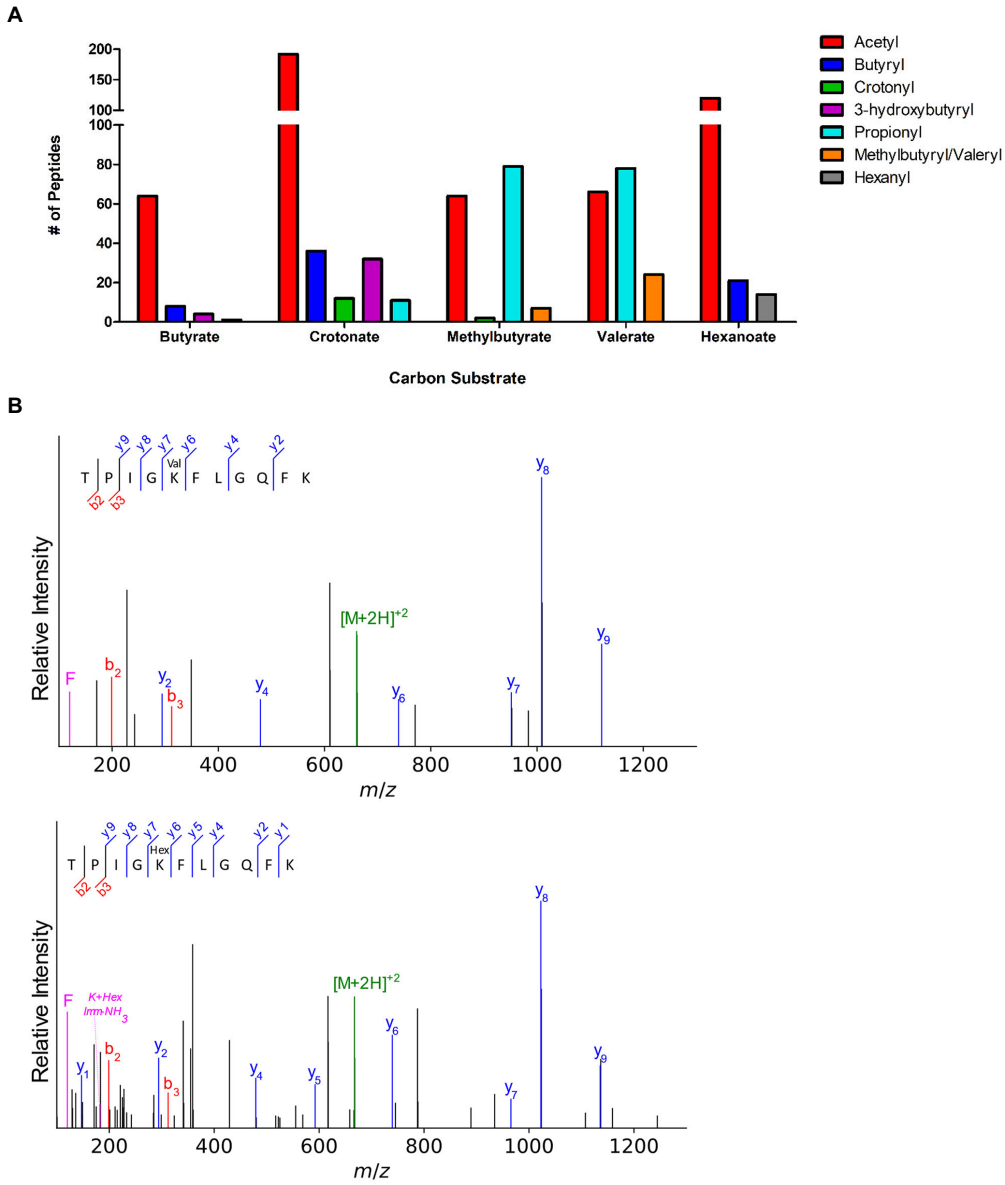
Novel acyl-lysine modifications in *S. wolfei* sub sp. *methylbutyratica*. **(A)** Acylated peptides across carbon substrates. **(B)** Tandem mass spectra of acetyl-CoA transferase (Ga0126451_1012) peptides show the 14 Da shift between valeryl- and hexanyl-lysine. Product ions (b-, y-, and immonium-related) are colored red, blue, and magenta, respectively; precursor ion is green.

To further explore syntrophic bacteria and acylation from RACS, we next investigated the acylome of *S. wolfei* subspecies *methylbutyratica*, a closely related syntroph that metabolizes longer carbon substrates. In addition to growth on butyrate and crotonate (C4), cells were also grown in *2-*methylbutyrate (C5), valerate (C5), and hexanoate (C6). Acyl-PTMs derived from RACS intermediates formed during substrate degradation were observed (Supplementary Table 2). Collectively, 353 acylated sites on 166 proteins, were identified. The types of acyl-modifications are similar to ones identified in *S. wolfei* Göttingen and in syntroph, *S. aciditrophicus* (Muroski et al., 2022), illustrating the similarity and large number of acylations present in syntrophic bacteria (Supplementary Table 4). The number and type of acyl PTMs observed across culture conditions is illustrated in Figure 6A.

In addition to the 4 types of acylation observed in *S. wolfei* Göttingen, longer acyl-modifications (methylbutyryl−/valeryl-, and hexanyl-lysine) were also observed, but only in cells grown on the corresponding carbon substrate (i.e., valeryl-lysine PTMs were observed in valerate grown cultures). Because methylbutyryl- and valeryl- are both five carbon chains (branched or linear), the two PTMs are indistinguishable by mass. They are grouped together in Figure 6A. The valeryl- and hexanyl-lysine modifications are the first identified in an organism. We verified the novel acyl-PTMs by comparing the tandem mass spectra and retention times to those from synthetic versions and sought acyl-lysine associated immonium ions in the tandem spectra (Figure 6B; Supplementary Figure 3; Muroski et al., 2021). Propionyl-lysine modifications were primarily observed in cells grown on odd chain carbon substrates (i.e., *2-*methylbutyrate and valerate) consistent with β-oxidative degradation that would produce propionyl-CoA in addition to acetyl-CoA from odd-carbon number acyl intermediates (Table 4; Supplementary Figure 4). Similarly, butyryl-lysine was not observed from cells cultivated on odd numbered carbon chain substrates and was only identified from even-carbon number substrates (butyrate, crotonate, and hexanoate) whose degradation produces butyryl-CoA or, for butyrate as substrate, make butyryl-CoA as the first step. The stark contrast in types of acyl-PTMs observed from different carbon sources suggests that the modifications directly correlate to the RACS produced by β-oxidation of the respective substrate.

**Table 4:** Acyl modified peptides identified in *S. wolfei* sub sp*. methylbutyratica*.

Sequence	Locus tag	Protein name	Modification site in peptide and type	Position in protein	Growth condition	Involved in beta-oxidation?
KDELEDVK	Ga0126451_10786	butyryl-CoA:acetate CoA-transferase	K1(Acetyl, Propionyl, Valeryl)	K49	Valerate	Yes
FSKAASLAK	Ga0126451_106150	butyryl-CoA dehydrogenase	K3(Acetyl, Butyryl, Hexanyl)	K314	Hexanoate	Yes
KALEDIAADQAIK	Ga0126451_106148	short chain enoyl-CoA hydratase	K1(Acetyl, Butyryl, Hexanyl)	K38	Hexanoate	Yes
EFGALGQKVFR	Ga0126451_10782	short chain enoyl-CoA hydratase	K8(Acetyl, Butyryl, Hexanyl)	K88	Hexanoate	Yes
KVVLYDIK	Ga0126451_1,013	*3-*hydroxyacyl-CoA dehydrogenase	K1(Acetyl, Propionyl, Valeryl)	K28	Valerate	Yes
DIKQEFVDR	Ga0126451_10783	*3-*hydroxyacyl-CoA dehydrogenase	K3(Acetyl, Propionyl, Valeryl)	K94	Valerate	Yes
GIAGIDKLLSK	Ga0126451_10783	*3-*hydroxyacyl-CoA dehydrogenase	K7(Acetyl, Propionyl, Valeryl)	K45	Valerate	Yes
TPIGKFLGQFK	Ga0126451_1012	acetyl-CoA C-acetyltransferase	K5(Acetyl, Propionyl, Valeryl)	K18	Valerate	Yes
TPIGKFLGQFK	Ga0126451_1012	acetyl-CoA C-acetyltransferase	K5(Hexanyl)	K18	Hexanoate	Yes
FKDEIVPVVIK	Ga0126451_10784; Ga0126451_11663	acetyl-CoA acetyltransferase /*3-*ketoacyl-CoA thiolase	K2(Acetyl, Propionyl, Valeryl)	K199	Valerate	Yes
FKDEIVPVVIK	Ga0126451_10784; Ga0126451_11663	acetyl-CoA acetyltransferase /*3-*ketoacyl-CoA thiolase	K2(Hexanyl)	K199	Hexanoate	Yes
ALELGVKPLMK	Ga0126451_106149	acetyl-CoA acetyltransferase	K7(Acetyl, Hexanyl)	K274	Hexanoate	Yes
AAEESKNEIIAK	Ga0126451_102211	ATP synthase F0 subcomplex B subunit	K6(Propionyl, Valeryl)	K132	Valerate	No
KITAESLGEAIK	Ga0126451_10799	Benzoyl-CoA reductase/2-hydroxyglutaryl-CoA dehydratase subunit, BcrC/BadD/HgdB	K1(Acetyl, Propionyl, Valeryl)	K195	Valerate	No
GKEVDVATADSK	Ga0126451_12425	acetate kinase	K2(Acetyl, Butyryl, Hexanyl)	K364	Hexanoate	No

Gene/protein locus tag destinations from the IMG JGI genome database.

We next investigated whether evidence of PTM crosstalk, or acyl-groups competing to modify the same residue, in metabolic enzymes was similar to that seen in the Göttingen subspecies. Several types of acyl groups modifying one residue were identified for 22 sites in 13 proteins, 10 of which are involved in β-oxidation (Table 4). The three other proteins were included because they had lysine residues modified with the novel valeryl- and hexanyl-lysine modifications, as well as other acyl-PTMs. The modifications observed shifted with changes in carbon source. For example, peptide FKDEIVPVVIK which is common to two proteins (Ga0126451_10784; Ga0126451_11663) both of which are an acetyl-CoA acetyltransferase/ *3-*ketoacyl-CoA thiolase had different acyl groups depending on the substrate. It was modified with acetyl- and hexanyl-lysines in hexanoate-grown cells, but contained acetyl-, propionyl-, and valeryl-lysine modifications from valerate-grown cells. This acetyl-CoA acetyltransferase is an ortholog of *S. wolfei* Göttingen Swol_2051 (95.7% sequence identity), which was also heavily acylated (Supplementary Figure 5). That acylated FKDEIVPVVIK (K199) is common to both *S. wolfei* subsp. *methylbutyratica* and *S. wolfei* Göttingen, demonstrates that certain acylation sites are conserved (Tables 3, 4). We also observed that, as the length of acyl-modification increases, so does peptide retention time, following previous reports of acylation effects on chromatography (Supplementary Figure 6; Moruz et al., 2012; Mizero et al., 2021). This chromatographic behavior should be carefully considered when performing proteomic studies investigating longer chain length modifications. For valeryl- and hexanyl-lysine modifications, there were several acyl-peptides identified at retention times that corresponded to unusually high percentages of organic phase (~80% acetonitrile), for MS proteomics. Changes in modifications with carbon substrates suggest that acyl-PTMs are driven by RACS, such that the presence or absence of a modification can act as a marker for the types of RACS produced. These metabolite-driven modifications demonstrate their intimate link to the cell’s metabolic activity.

### Crystal structure of *Syntrophomonas wolfei* Göttingen acetyl-CoA acetyltransferase reveals potential impacts of acylation

**Figure 7:**
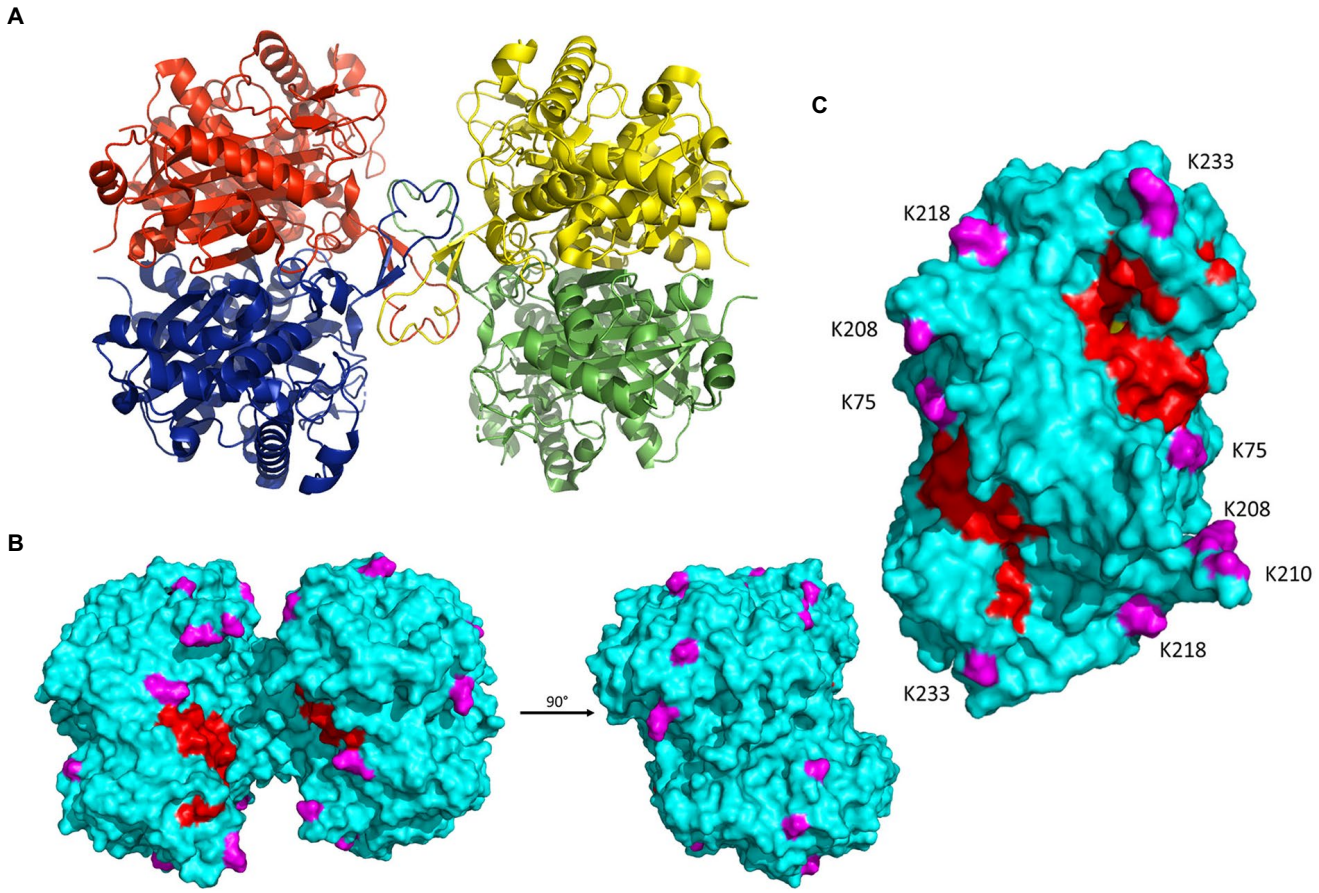
Crystal structure of *S. wolfei* Göttingen acetyl-CoA transferase, Act2 (Swol_0675; PDB entry: 7N7Z; 2.02 Å resolution; R-free: 0.274; R-work: 0.233; R-observed 0.237). **(A)** Act2 tetramer with monomers colored individually. **(B)** Front (same orientation as A) and side views of the molecular surface of the Act2 tetramer. Acylated residues identified from proteomic datasets are highlighted in magenta; covering and pantetheine loop are colored in red. **(C)** Side view of the molecular surface of the Act2 homodimer, with similar coloring scheme as B. Figures were generated with PyMOL (Anon, n.d.). Table 1 lists additional data collection and structural refinement statistics.

*Syntrophomonas wolfei* Göttingen uses acyl-CoA transferases to activate fatty acid substrates by synthesizing the respective CoA derivative. An acyl-CoA transferase paralog of particular interest is Act2 (Swol_0675). Act2 was more abundant than other Act paralogs, all of which did not change significantly between growth conditions. Act2 had numerous acylations and certain sites were modified by several different acyl groups (Table 3). To understand how these PTMs might affect the enzyme’s function, the structure of Act2, encoded by the gene Swol_0675, was determined by X-ray crystallography (Figure 7A; Table 1). The asymmetric unit of the tetragonal crystal lattice contains two molecules of Act2, which form a homodimeric structure. About 15% of the solvent accessible surface area of the monomer, or ~ 15,000 Å^2^, is buried in the intermolecular interface. Examination of crystal packing indicates that the Act2 homodimer likely forms a homotetramer mediated by a short β-strand-containing loop spanning residues Tyr127 to Asp140. A homotetrameric structure is consistent with the Act2 molecular mass estimated from size exclusion chromatography, with the computational prediction by the PISA webserver (Krissinel and Henrick, 2007), and with existing acetyl-CoA transferase structures that also have a similar homotetrameric structures. With this structure, we were able to visualize where the modified residues were located.

Acetyl-CoA transferases from a large variety of organisms have been structurally characterized and a search for enzymes structurally related to Act2 using the PDBeFold server (Krissinel and Henrick, 2004) allowed us to identify conserved catalytic regions in *S. wolfei* Act2 shared amongst Gram-negative and Gram-positive bacteria. The three most closely related structures are the Act structures from *Escherichia coli* (PDBid 5F38, Z-score 20.5, rmsd of 1.04 Å over 386 amino acids with sequence identity of 46%), *Clostridium difficile* (PDBid 4DD5, Z-score 19.8, rmsd of 1.05 Å over 384 amino acids with sequence identity of 48%), and *Clostridium acetobutylicum* (PDBid 4WYR, Z-score 19.7, rmsd of 1.05 Å over 386 amino acids with sequence identity of 46%; Supplementary Figure 7). Several regions are conserved among the four structures including the CoA binding region near the active site and the substrate tunnel. The position of the four key catalytic residues (C91, N319, H357, C387) is virtually identical in all four of the structures as is the shielding of the active site by the covering (residues 146–161) and pantetheine loops (residues 246–250), which were identified in the human mitochondrial acetoacetyl- CoA thiolase (T2; Haapalainen et al., 2007; Supplementary Figure 8).

All the acyl-lysine sites identified in our proteomic datasets were mapped onto the *S. wolfei* Act2 structure and all are on the protein’s surface, with three sites located closer to the covering and pantetheine loop, K75, K208, and K233 (Figures 7B,C). The position of acetylated residue K75 is conserved in the *E. coli* and *C. acetobutylicum* Act structures (substituted with glycine in *C. difficile* sequence) and the position of K233 is conserved in all four structures (although disordered in the *E. coli* structure). Residues K208 and the highly conserved K233 were found to be acetylated and/or butyrylated in *C. acetobutylicum* as found in *S. wolfei* Göttingen Act2 (Xu et al., 2018). *C. acetobutrylicum* can also generate butyryl-CoA from acetyl-CoA with homologous β-oxidation enzymes. These shared sites of modification and production of similar RACS indicates that lysine acylation may also be conserved. The proximity of these modified lysine residues near the substrate tunnel suggest that these PTMs may be connected to the local buildup of RACS in this region. While the other modification sites are located on the enzyme’s surface but not near the substrate tunnel, these lysine residues could be involved in salt-bridge interactions that mediate tetramer formation. If these interactions are present, acylation at these residues could potentially be disruptive for protein–protein interactions.

To further investigate this relationship between RACS and acyl-PTMs, we performed *in vitro* acylation assays with Act2, characterizing the sites of acylation to determine whether modified sites could be recapitulated non-enzymatically and provide insight into their potential *in vivo* regulation. Previous studies have demonstrated that *in vitro* incubation of proteins with RACS can give rise to non-enzymatic lysine acylation (Wagner and Payne, 2013; Parks and Escalante-Semerena, 2020; Baldensperger and Glomb, 2021), but the likelihood of lysine modification on a per residue basis in Act2 is largely unexplored. Factors such as local RACS concentrations and protein–protein interactions affecting site accessibility may impact lysine modification *in vivo.* Purified recombinant *S. wolfei* Göttingen Act2 was incubated either with acetyl-CoA or butyryl-CoA and lysine modifications were detected *via* LC–MS/MS. Out of the 25 lysine residues in Act2, 23 sites are located on the surface and are solvent-accessible. We detected 12 sites that were acylated *in vitro* and 5 of these sites matched sites that were found to be acylated in the proteomic datasets (Table 5). The lysine modifications identified *in vitro* are similar to those found in *S. wolfei in vivo,* indicating that a buildup of RACS can, indeed, yield non-enzymatic lysine acylations. Out of these 5 sites, K75, K208, and K304 were located near the covering and pantetheine loops that surrounds the substrate tunnel (Figure 7C), suggesting that these acyl modifications could affect substrate accessibility. We also observed lysine modifications that were either found only *in vivo* or *in vitro* which highlights the possibility that these modified sites are regulated *in vivo* (Table 5). We also observed that a majority of these residues were readily modified by acetyl-CoA, but to see any butyryl modifications required a 50-fold increase in butyryl-CoA concentration (1 μM acetyl-CoA versus 50 μM butyryl-CoA). The large differences in acyl-CoA concentrations required for *in vitro* modification indicates that these RACS have different propensities for lysine modification (Simic et al., 2015).

**Table 5:** Sites of lysine acylation in *S. wolfei* Göttingen acetyl-CoA transferase Act2 (Swol_0675).

Lysine position	Where located?	Modified *in vivo*?	Modified *in vitro*?
75	Surface	Acetyl, Butyryl, Crotonyl, *3-*Hydroxybutyryl	Acetyl
109	Interior	No	No
141	Tetramerization loop	No	No
170	Surface	No	No
191	Surface	Acetyl	No
208	Surface	Acetyl, Butyryl	Butyryl
210	Surface	No	Acetyl
211	Surface	No	Acetyl
214	Surface	Acetyl	Acetyl, Butyryl
218	Surface	Acetyl	Acetyl
233	Surface	Acetyl, Butyryl	No
239	Surface	No	No
240	Surface	No	No
264	Surface	No	Acetyl
265	Surface	No	Acetyl
266	Surface	No	Acetyl
273	Surface	No	Acetyl
277	Surface	No	Acetyl
289	Surface	No	No
301	Interior	No	No
304	Surface	Acetyl	Acetyl
309	Surface	No	No
334	Surface	No	No
345	Surface	No	No
381	Surface	Acetyl	No

Full sequence of Act 2 is in Supplementary Figure S6.

## Discussion

Our systems-level characterization of lysine acylation in *S. wolfei* Göttingen and subsp. *methylbutyratica* has demonstrated a relationship between RACS found in the β-oxidation pathway and acyl-lysine PTMs. The acylome profiles of cells grown under different carbon substrates differ not only in the protein sites modified and the type of acylation, but also the relative abundance of modifications. We also identified novel acyl-modifications (valeryl- and hexanyl-lysine), present only when cells were fed the respective substrate, and observed that the acyl-PTMs displayed reflect the distinct RACS produced when degrading odd- or even-chain fatty acids. These changes in the proteins’ modification, especially for metabolic enzymes whose abundances remain stable between changing growth conditions, suggests that *S. wolfei* may be regulating its metabolism post-translationally. This means of metabolic regulation has been suggested in other syntrophs (Supplementary Table 3 Muroski et al., 2022). Because syntrophic bacteria live in energy limited conditions, such modifications could reduce protein turnover and reduce the cell’s reliance on energetically costly protein synthesis.

In the cell, modifications could act as a metabolic brake, as they have been shown to reduce enzymatic activity (Crosby et al., 2010). Our structural studies of *S. wolfei* Göttingen acetyl-CoA transferase, Act2, indicate that modified residues are located near catalytically critical regions of the protein, suggesting that fluctuations in modification levels may alter the activity of metabolic enzymes. In removing charges from the surface of the proteins, acylations could also influence protein–protein interactions; e.g., to speed product removal by making certain conversions more favorable. Notably, many of the modified enzymes we found form complexes with themselves or other enzymes (Crable et al., 2016), and these types of interactions have been demonstrated to be altered by lysine acylations in other systems (Lin et al., 2015; El Kennani et al., 2018; Zhu et al., 2019). While many of the observed acylations are structurally similar, differing by only a methyl or hydroxy group in some cases, such slight differences are sufficient to alter deacylase specificity and activity significantly (McClure et al., 2017). These acyl-PTMs could act as an efficient mechanism to fine-tune catabolic pathways. Table 2 reveals that cells grown in crotonate showed increases in both modified sites and proteins for acetylation and *3-*hydroxybutyrylation, but not butyrylation or crotonylation. It could be imagined that *3-*OH-butyryl CoA might accumulate under crotonate culture, given that its oxidation to acetoacetyl-CoA is thermodynamically unfavorable. In contrast, the thermodynamic favorability of reducing crotonate to butyrate makes butyryl-CoA accumulation less likely. Increased growth under crotonate cultivation may drive acetylation. Acetyl phosphate is a potent acetylator, as is evident when comparing relative acetylation levels in *S. wolfei* and *S. aciditrophicus* (Supplementary Table 4). Lacking an acetate kinase, *S. aciditrophicus* does not produce acetyl phosphate and consequently displays much lower levels of acetylation than *S. wolfei*.

One could also speculate that metabolite-induced modifications reducing enzymatic activity might facilitate a switch between two pathways. In principle, while metabolizing crotonyl-CoA during exponential phase, *S. wolfei* pure cultures might, whenever NADH levels are high, divert production of L(+)-*3*-hydroxybutyryl-CoA destined for oxidation to acetoacetate (and NADH production) to synthesizing D(−)-*3-*hydroxybutyryl-CoA for polymerization into poly-beta-hydroxybutyrate (PHB). A temporary switch to store carbon/energy as PHB could avoid an immediate increase in NADH levels (reductive stress). Once the NADH/NAD^+^ ratio decreased, an NAD^+^-dependent sirtuin could reverse acyl modifications, switching back to acetoacetate production.

Implementing acyl-PTMs as a negative feedback inhibitor could reduce carbon and reductive stress. This means of metabolic regulation could be useful for pathways, like β-oxidation, that incorporate reversible steps. This flexibility in pathway directionality is a phenomenon observed in other syntrophic bacteria (James et al., 2019). When cellular reducing power is excessive, the cell might acylate NAD-dependent metabolic enzymes like *3-*hydroxybutyryl CoA dehydrogenase Swol_2030, which was found to be acylated, to slow the production of NADH. High levels of NADH would lead to increased H_2_ partial pressure, which could make further metabolism energetically difficult. Should these NAD-dependent metabolic enzymes be acylated, however, both the upstream and downstream reactions would slow, until NAD^+^ could be regenerated by other means. In syntrophs, NADH reoxidation by ferredoxin-independent hydrogenases or formate dehydrogenases can only proceed if H_2_ partial and formate levels are low and a methanogenic partner is needed to maintain these levels (Schink, 1997; Losey et al., 2017; Agne et al., 2022). Acyl-CoA dehydrogenases Swol_2126 and _2030 had increased acylation in crotonate monocultures, suggesting that higher acylation levels may indeed be a response to the lack of a methanogenic partner. Furthermore, one can envision that changes in *S. wolfei* acylation profiles in cocultures may be a response to changes in the methanogen’s metabolism. If hydrogen consumption by the methanogenic partner is altered, NADH reoxidation in syntrophs will be affected, changing the flux of its NAD-dependent metabolic pathways and resulting in a buildup of RACS.

As these metabolite driven acyl-modifications impact metabolic activity, deacylases such as sirtuins, regulate the PTMs. Sirtuins are NAD^+^ dependent enzymes that remove a variety of acyl-lysine modifications. Bacterial sirtuins with promiscuous deacylase activities have been identified in model systems such as *E. coli* and in another syntroph, *S. aciditrophicus* (Zhao et al., 2004; Bheda et al., 2016; Muroski et al., 2022). Sirtuins have been linked to stress resistance mechanisms related to oxidative stress and carbon starvation (Ma and Wood, 2011; Abouelfetouh et al., 2015). Sirtuin activity also depends on NAD^+^, linking lysine acylation to the cellular redox state. Sirtuins may be active in this system as the uneven distribution of acylation abundances in the proteome and *in vitro* acylation of Act2 data both suggest that these PTMs are regulated. By sequence homology, we identified a putative membrane-bound sirtuin, Swol_1033, in *S. wolfei* Göttingen. Characterizing its substrate specificity and activity remain an important direction for future studies of this bacterium.

To further understand the metabolic impact of lysine acylation in syntrophs and other organisms, we should consider characterizing them in a comprehensive and unbiased manner. Mass spectrometry-based proteomics is a powerful tool for analyzing the acylome (Xu et al., 2022). Proteomics can identify the broad scope of PTMs occurring within a biological system, but analyzing a wide range of acylations can present analytical challenges such as potential sequence misidentifications (Lee et al., 2013; Kim et al., 2016). The ambiguities from isomeric and/or isobaric combinations must be considered while assigning modified residues in proteomic datasets, especially when characterizing PTM crosstalk on multiply modified peptides. Incorporated diagnostic marker ions of lysine acylation in proteomics analyses can help increase confidence in the PTMs identified, particularly when peptides contain multiple acyl modifications (Supplementary Figure 2; Muroski et al., 2021).

In order to better understand how multiple acyl-PTMs relate to one another in *S. wolfei*, MS techniques such as middle-down and top-down proteomics may be useful for characterizing PTM crosstalk (Leutert et al., 2021). Unlike bottom-up proteomics, which was performed in this study and digests proteins into peptides, middle-down proteomics utilizes different digestion strategies to obtain partially digested proteins. This approach generates longer peptides, increasing the likelihood of observing co-occurring PTMs. Top-down proteomics does not incorporate any protein digestion and instead measures whole proteins. This technique provides proteoform level information, allowing one to identify PTM combinations present on a protein regardless of their sequence proximity. There remain significant challenges, however, in top-down proteomics as it is limited by low throughput, low proteome coverage, and the complexity of acylations adds great ambiguity to the data interpretation. To characterize PTM crosstalk, one must consider the combinatorial nature of these acyl modifications on multiple potential sites which generates large numbers of theoretical proteoforms for consideration and computational PTM assignments must be carefully inspected (Moradian et al., 2014; Zhou et al., 2017). It is also possible that unknown modifications might be missed. Further investigation of these acyl-PTMS in combination with each other is important to fully understanding how these critical environmental microbial consortia regulate their metabolism. More broadly, additional studies of acyl-PTMs in other bacteria may further uncover the intricate link between RACS intermediates and its impact on metabolic regulation.

## Data availability statement

Mass spectrometry data have been deposited to the ProteomeXchange Consortium (proteomexchange.org) via the MassIVE partner repository with the dataset identifier PXD034881. MS Skyline files have been deposited to Panorama and can be accessed from: https://panoramaweb.org/JGqmrC.url. Crystallographic data, refinement statistics, and the structure has been deposited in the Protein Data Bank under accession code 7N7Z.

## Author contributions

JF, JM, MM, RG, RL, and JL designed research. JF, JM, MA, JS, and NW performed research. JF, JM, MA, JS, NW, MM, RL, and RG analyzed data. JF, JM, MA, MM, RG, RL, and JL wrote and edited the paper. All authors contributed to the article and approved the submitted version.

## Funding

Funding from the U.S. Department of Energy (DOE) Office of Science (BER) contract DE-FC-02-02ER63421 (to JL and RG; UCLA/DOE Institute for Genomics and Proteomics), NIH Ruth L. Kirschstein National Research Service 18 Award (to JF; GM007185), NSF Awards 1515843 and 1911781 to RG and MM, and NSF Graduate Research Fellowship (to JF; DGE-1650604) are acknowledged. A component of this research was conducted at the Protein Expression Technology Center of the UCLA-DOE Institute for Genomics and Proteomics which is supported by the United States. DOE Office of Science, (BER) program under Award Number DE-FC02-02ER63421. Diffraction data was collected at the Northeastern Collaborative Access Team beamlines, which are funded by the National Institute of General Medical Sciences from the National Institutes of Health (P30 GM124165). This research used resources of the Advanced Photon Source, a US DOE Office of Science User Facility operated by Argonne National Laboratory under Contract No. DE-AC02-06CH11357.

## Conflict of interest

The authors declare that the research was conducted in the absence of any commercial or financial relationships that could be construed as a potential conflict of interest.

## Publisher’s note

All claims expressed in this article are solely those of the authors and do not necessarily represent those of their affiliated organizations, or those of the publisher, the editors and the reviewers. Any product that may be evaluated in this article, or claim that may be made by its manufacturer, is not guaranteed or endorsed by the publisher.

## Footnotes

^1^ https://panoramaweb.org/JGqmrC.url

## Abbreviations

ABC: ammonium bicarbonate
AGC: automatic gain control
BC: butyrate coculture
CoA: Coenzyme A
CM: crotonate monoculture
DDA: data-dependent acquisition
DTT: dithiothreitol
eFASP: enhanced filter-assisted sample preparation
HCD: high-energy collisional dissociation
HILIC: hydrophilic interaction chromatography
IAM: iodoacetamide
KEGG: Kyoto Encyclopedia of Genes and Genomes
LC–MS/MS: liquid chromatography tandem mass spectrometry
MS: mass spectrometry
MWCO: molecular weight cut-off
NAD+/NADH: nicotinamide adenine dinucleotide (oxidized/reduced)
NE-CAT: Northeastern Collaborative Access Team
PEG: polyethylene glycol
PHB: poly-beta-hydroxybutyrate
PRM: parallel reaction monitoring
PTMs: post-translational modifications
RACS: reactive-acyl Coenzyme A species
