# Supplementary material for: Dynamic acylome reveals metabolite driven modifications in Syntrophomonas wolfei
Source: Front Microbiol. 2022 Nov 7;13:1018220. doi: 10.3389/fmicb.2022.1018220 (PMC9676460; doi:10.3389/fmicb.2022.1018220)
Supplement: Supplementary file 1 [file Data_Sheet_1.docx]

**Dynamic acylome reveals metabolite driven modifications in *Syntrophomonas wolfei***

**Janine Y. Fu^1^, John M. Muroski^1^, Mark A. Arbing^5^, Jessica A. Salguero^1^, Neil Q. Wofford^2^, Michael J. McInerney^2^, Robert P. Gunsalus^3,5,6^, Joseph A. Loo^1,4,5,6^, Rachel R. Ogorzalek Loo^1,5,6*^**

^1^Department of Chemistry and Biochemistry, University of California, Los Angeles, CA, USA

^2^Department of Microbiology and Plant Biology, University of Oklahoma, Norman, Oklahoma, USA

^3^Department of Microbiology, Immunology and Molecular Genetics, University of California, Los Angeles, Los Angeles, California, USA

^4^David Geffen School of Medicine, Department of Biological Chemistry, University of California, Los Angeles, CA, USA

^5^UCLA-DOE Institute, University of California, Los Angeles, CA, USA

^6^UCLA Molecular Biology Institute, University of California, Los Angeles, CA, USA

*** Correspondence:**Rachel R. Ogorzalek Loo
rloo@mednet.ucla.edu

Keywords: syntrophy, proteomics, mass spectrometry, post-translational modifications, lysine acylation, *Syntrophomonas wolfei*

Supplementary Material

# Supplementary Tables

**Supplemental Table 1.** Ions targeted in PRM experiments for quantitative analysis

(Please refer to attached Excel spreadsheet.)

**Supplemental Table 2.** Acyl modifications searched for in proteomic datasets

| Acyl Modification | Chemical Formula | Monoisotopic Mass Shift | Average Mass Shift | Immonium Ion | Cyclized Immonium Ion |
| --- | --- | --- | --- | --- | --- |
| Acetyl | C_2_H_2_O | 42.01056 | 42.03677 | 143.1179 | 126.0913 |
| Butyryl | C_4_H_6_O | 70.04186 | 70.09001 | 171.1492 | 154.1226 |
| Crotonyl | C_4_H_4_O | 68.02621 | 68.07413 | 169.1336 | 152.107 |
| *3-H*ydroxybutyryl | C_4_H_6_O_2_ | 86.03678 | 86.08942 | 187.1441 | 170.1176 |
| Acetoacetyl | C_4_H_4_O_2_ | 84.02113 | 84.07353 | 185.1285 | 168.1019 |
| Valeryl | C_5_H_8_O | 84.05751 | 84.11663 | 185.16475 | 168.13821 |
| Pentenoyl | C_5_H_6_O | 82.04186 | 82.10075 | 183.14916 | 166.12256 |
| *3-H*ydroxypentanoyl | C_5_H_8_O_2_ | 100.0524 | 100.116 | 201.1597 | 184.1331 |
| *3-*Oxopentanoyl | C_5_H_6_O_2_ | 98.03678 | 98.10016 | 199.14408 | 182.11748 |
| Propionyl | C_3_H_4_O | 56.0262 | 56.06339 | 157.1266 | 140.1069 |
| Hexanoyl | C_6_H_10_O | 98.07316 | 98.14326 | 199.18046 | 182.15386 |
| Hexenoyl | C_6_H_8_O | 96.05751 | 96.12737 | 197.16481 | 180.13821 |
| *3-*Hydroxy-Hexanoyl | C_6_H_10_O_2_ | 114.0681 | 114.1427 | 215.1754 | 198.1488 |
| *3-*Oxohexanoyl | C_6_H_8_O_2_ | 112.0524 | 112.1268 | 213.1597 | 196.1331 |

**Supplemental Table 3.** Proteins and peptides identified in *S. wolfei* Göttingen and *S. wolfei* sub sp. *methylbutyratica,*

(Please refer to attached Excel spreadsheet.)

**Supplemental Table 4.** Unique acyl peptides identified in proteomic studies of *S. wolfei* Göttingen*,* *S. wolfei* sub sp. *methylbutyratica,* and *Syntrophus aciditrophicus.*

| **# of Unique Acyl Peptides** | ***S. wolfei*** **Göttingen** | ***S. wolfei* sub sp. *methylbutyratica*** | ***S. aciditrophicus* SA** |
| --- | --- | --- | --- |
| Acetyl | 506 | 300 | 120 |
| Butyryl | 113 | 51 | 3 |
| *3-*Hydroxybutyryl | 55 | 34 | 6 |
| Crotonyl | 15 | 16 | 2 |
| Propionyl | *N.A.* | **123** | *N.A.* |
| Methylbutyryl/Valeryl | *N.A.* | **20** | *N.A.* |
| Glutaryl | *N.A.* | *N.A.* | **10** |
| Hexanyl | *N.A.* | **14** | *N.A.* |
| Benzoyl | *N.A.* | *N.A.* | **2** |
| *3-*Hydroxypimelyl | *N.A.* | *N.A.* | **19** |

Numbers of acyl peptides identified are summed across all carbon sources investigated in this study and in *Muroski et al.*(Muroski et al., 2021)*.* *N.A.* denotes modifications not searched for in the proteomic datasets, because they were unrelated to RACS produced by that organism under the conditions investigated. Acyl modification types unique to the indicated strain are in bold.

# Supplementary Figures


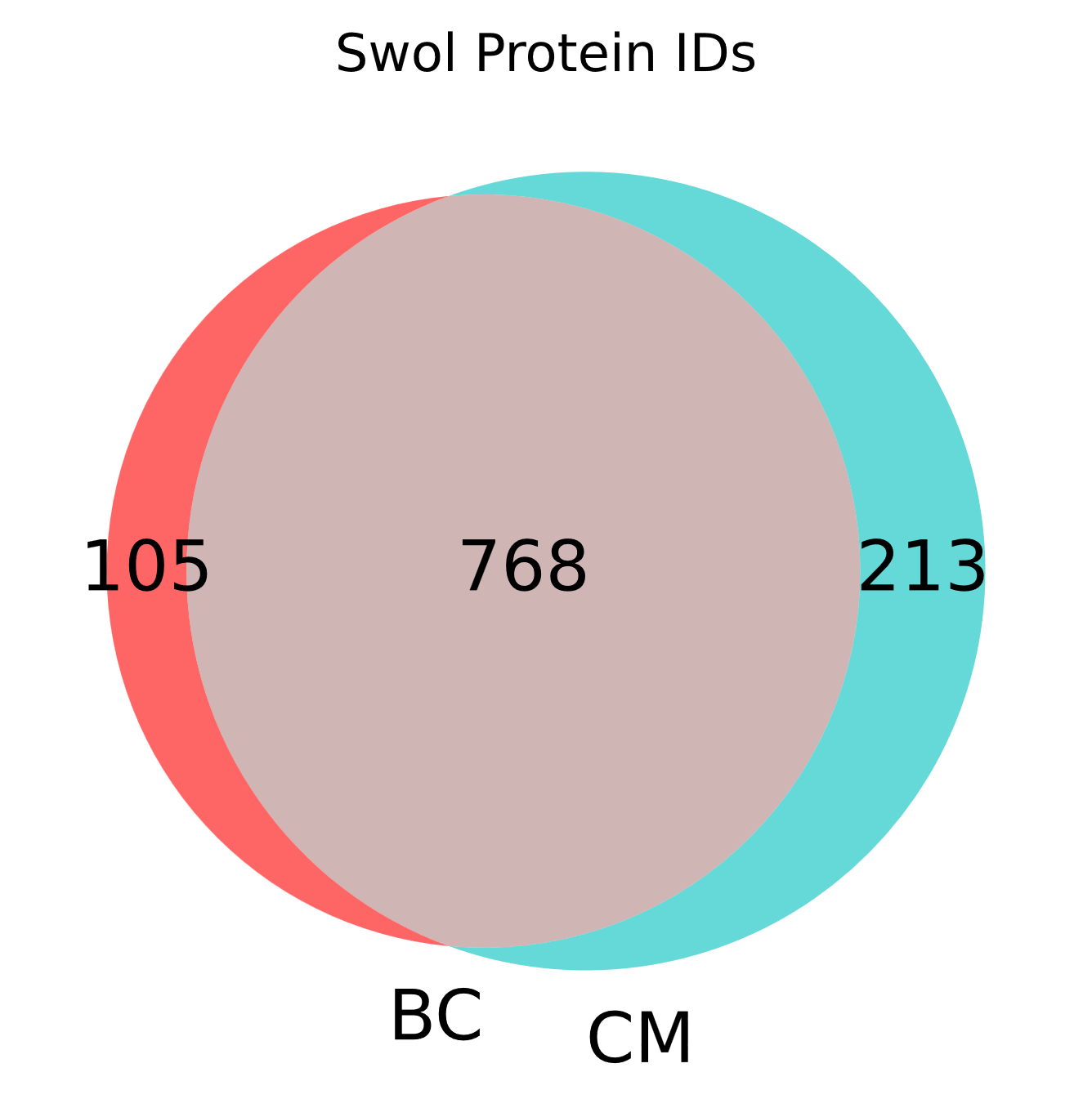


**Supplemental Figure S1. Numbers of proteins identified in butyrate cocultures (BC) and crotonate monocultures (CM) from HILIC-fractionated lysates of *S. wolfei* Göttingen.**

**
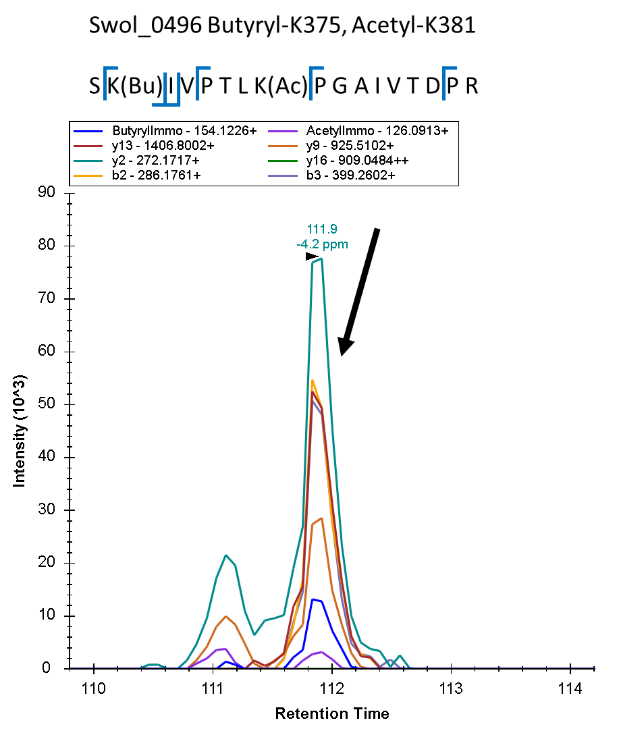

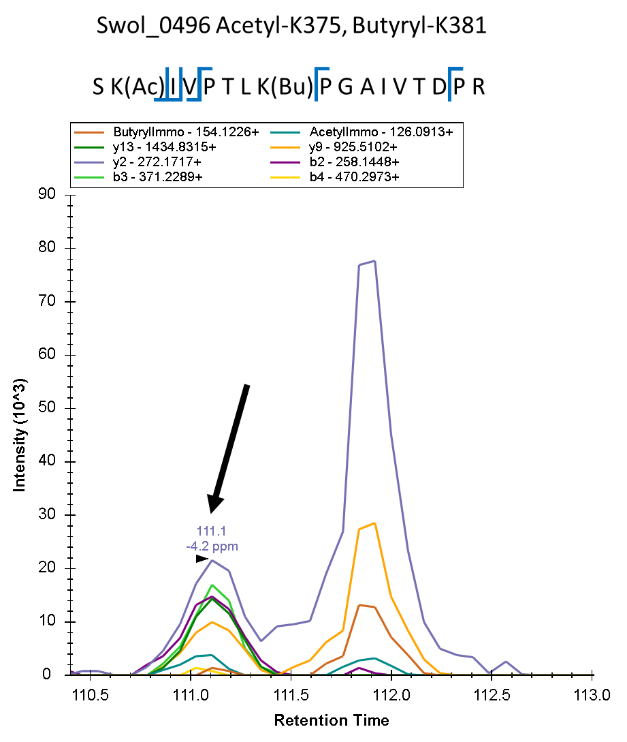

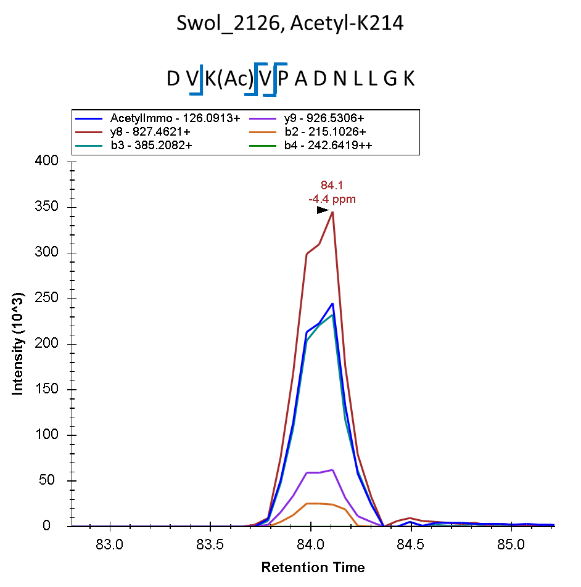

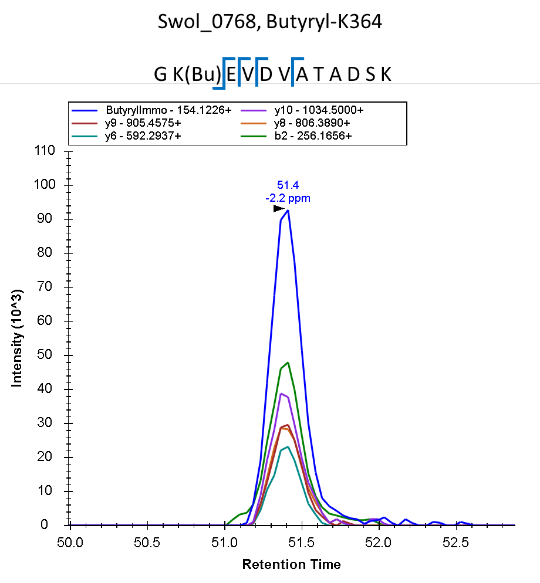

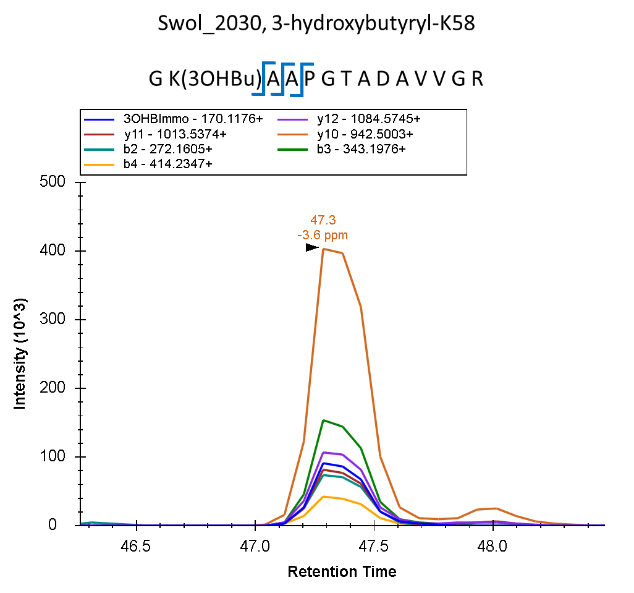
**

**E**

**D**

**C**

**B**

**A**

**Supplemental Figure S2.** **Skyline traces of MS/MS level quantitation of *S. wolfei* Göttingen acyl-peptides incorporating diagnostic immonium ions.** **A-C)** Extracted MS/MS ion chromatograms for DVK(Ac)VPADNLGGK (Swol_2126), GK(Bu)EVDVATADSK (Swol_0768), and GK(3OHBu)AAPGTADAVVGR (Swol_2030), respectively. Traces corresponding to each acyl-lysine immonium ion are blue. **D-E)** Elution profiles of extracted MS/MS ion chromatograms for Swol_0496 acyl-peptide isoforms SK(Ac)IVPTLK(Bu)PGAIVTDPR and SK(Bu)IVPTLK(Ac)PGAIVTDPR. Traces for the acyl-lysine immonium ions confirms that both acetyl- and butyryl-lysine modifications are present in the peptides. Retention time is in minutes.

**A**


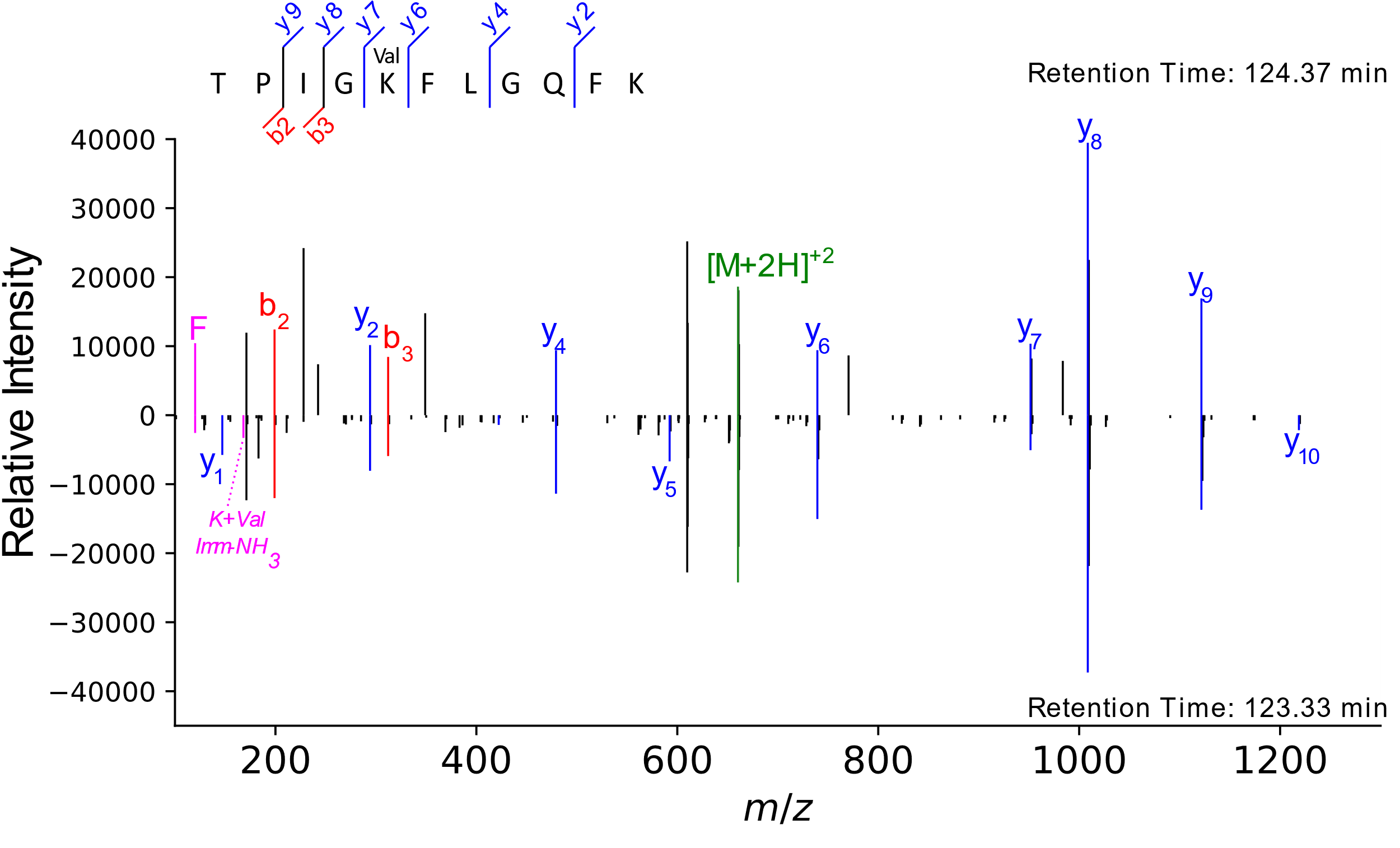

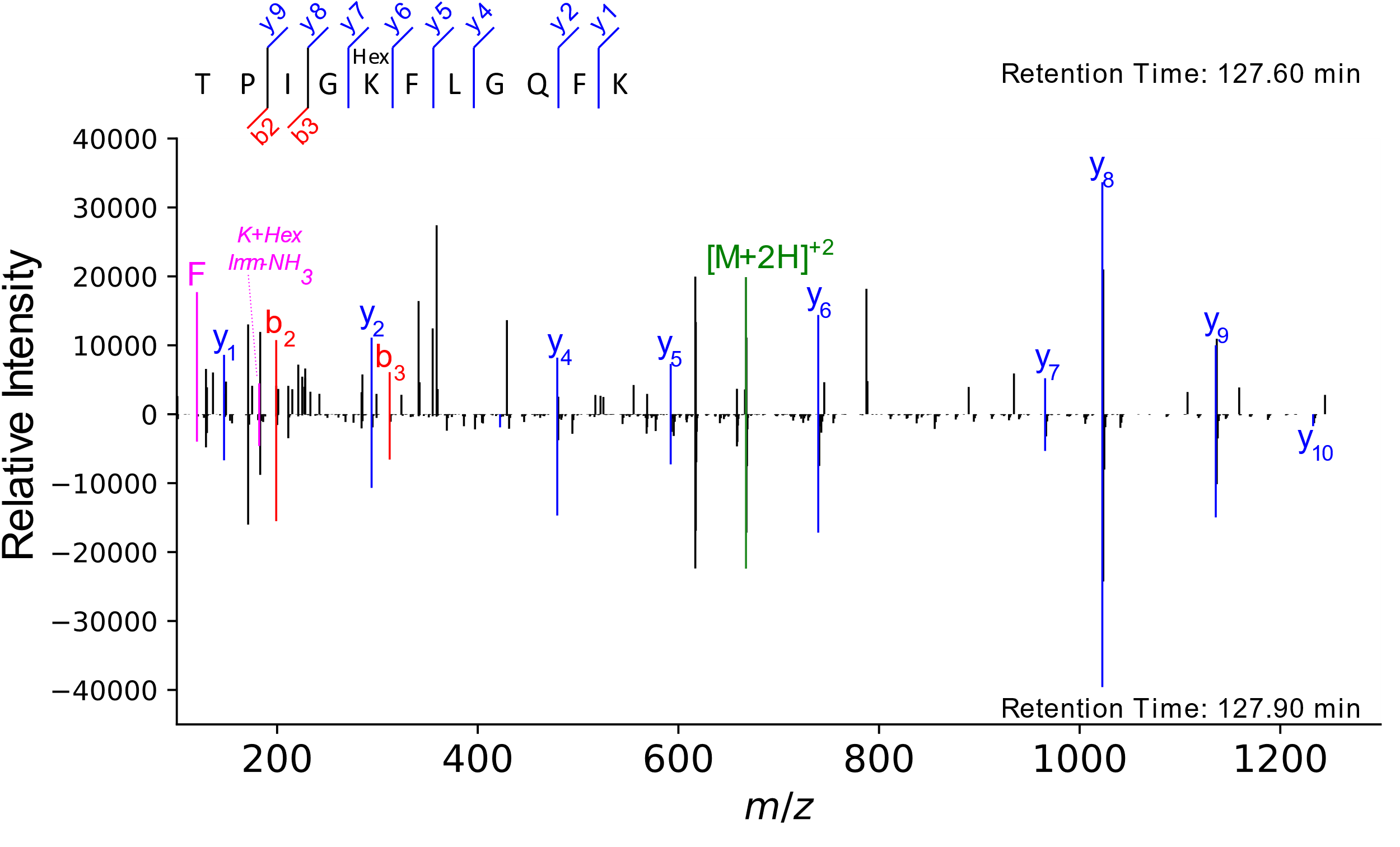


**B**

**Supplemental Figure S3.** **Comparing valeryl- and hexanyl-lysine modified peptides from in *S. wolfei* sub sp. *methylbutyratica* to synthetic standards*.* A-B)** MS/MS spectra TPIG**K**FLGQFK from acetyl-CoA transferase (Ga0126451_1012) with valeryl- and hexanyl-lysine modifications on the bolded lysine. Upper spectra are from peptides identified *in vivo* and lower spectra are from acylated reference peptides. Acyl-lysine immonium ions are highlighted in pink; b- and y-product ions are in red and blue; the precursor ion is green.


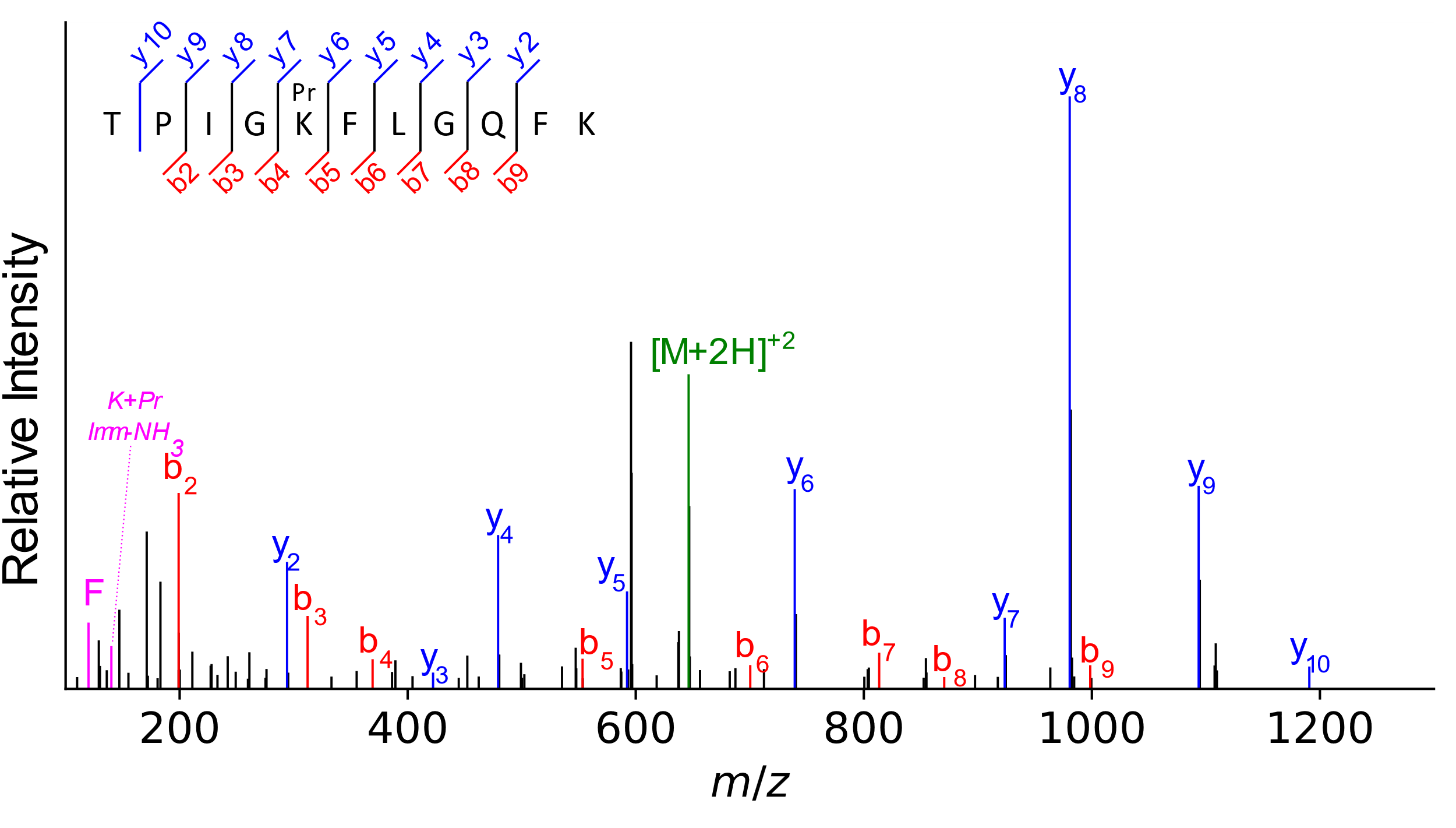


**Supplemental Figure S4. Propionyl-lysine modifications in *S. wolfei* sub sp. *methylbutyratica*.** Fragmentation spectra (MS/MS) showing mass shift corresponding to propionyl-lysine modifications on acetyl-CoA transferase (Ga0126451_1012). Product ions (b-, y-, and immonium-related) are colored in red, blue, and magenta, respectively; precursor ion is green.


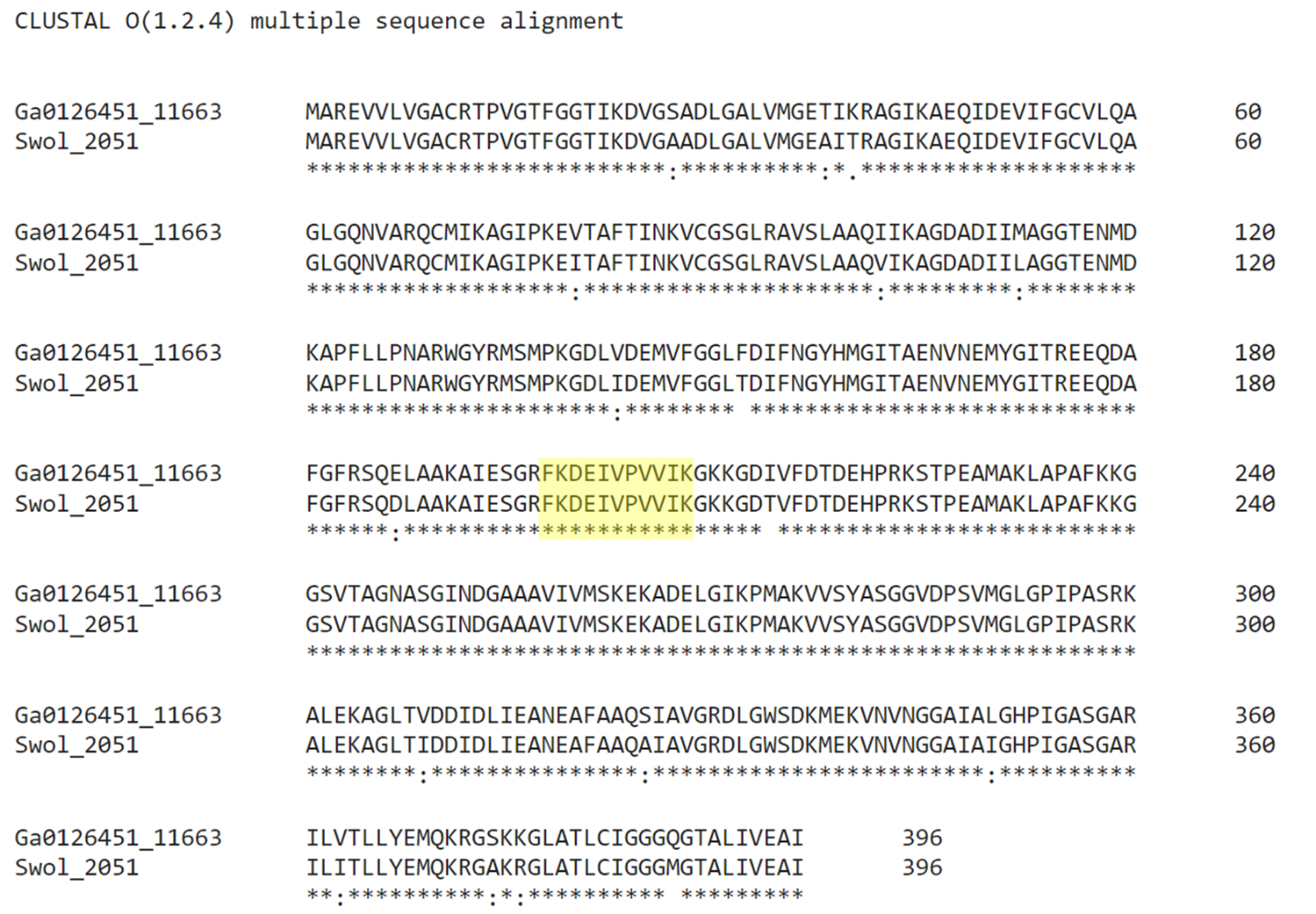


**Supplemental Figure S5.** **CLUSTAL Sequence alignment of acetyl-CoA acetyltransferases from *S. wolfei* sub sp. *methylbutyratica* and *S. wolfei* Göttingen*.*** Alignment of *S. wolfei* sub sp. *methylbutyratica* (Ga0126451_1153) to *S. wolfei* Göttingen Swol_2051. There is 95.7% sequence identity. The acylated peptide spanning residues 198-208, F**K**DEIVPVVIK (AcK199), identified in proteomic data of both *S. wolfei* sub species is highlighted in yellow.


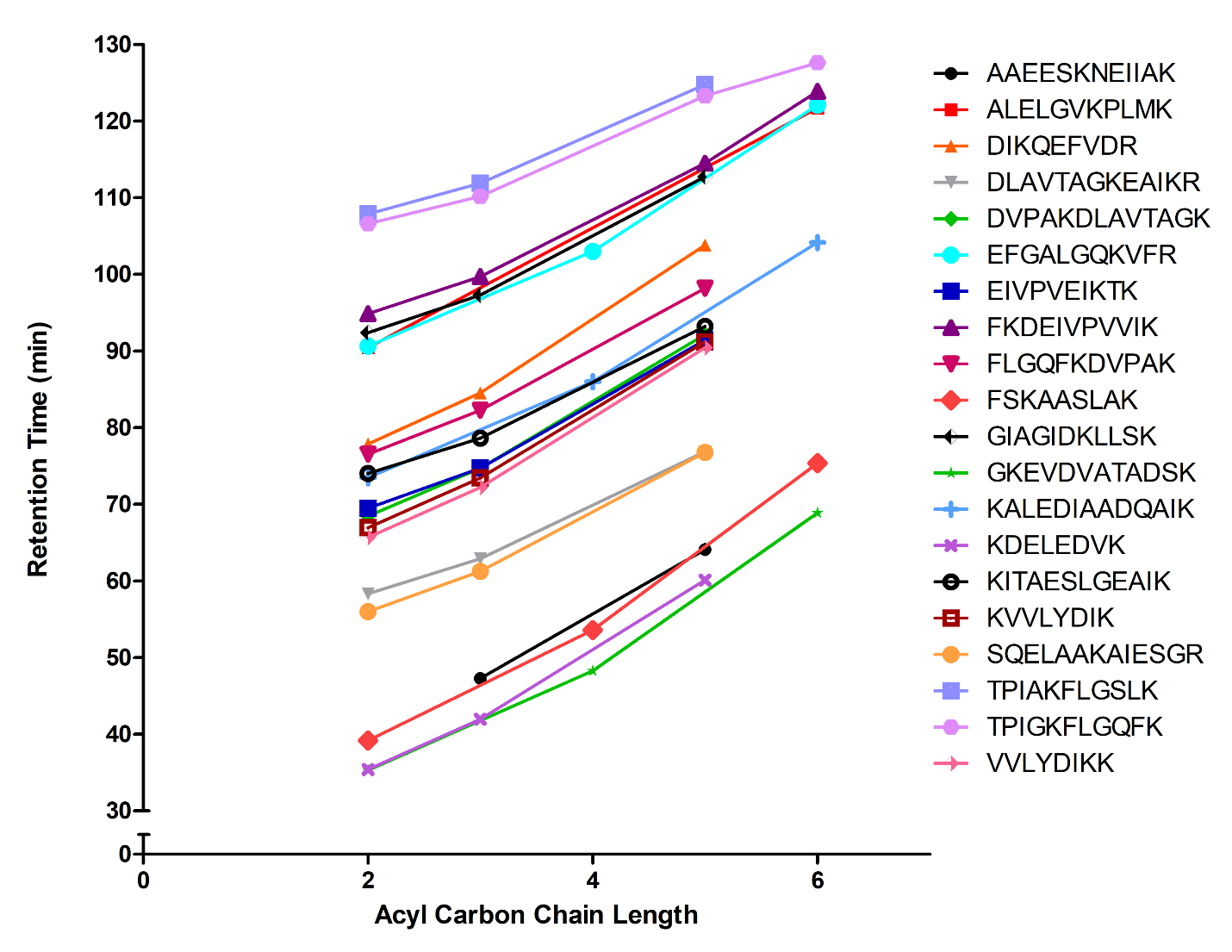


**Supplemental Figure S6. Comparison of acyl-peptide liquid chromatography retention time *versus* acyl-PTM carbon chain length.** Twenty acyl-peptides from *S. wolfei* sub sp. *methylbutyratica* were found to be modified with at most 4 types of acyl-groups. Carbon chain lengths of 2, 3, 4, 5, and 6 correspond to acetyl-, propionyl-, butyryl-, valeryl-, and hexanyl-lysine modifications.


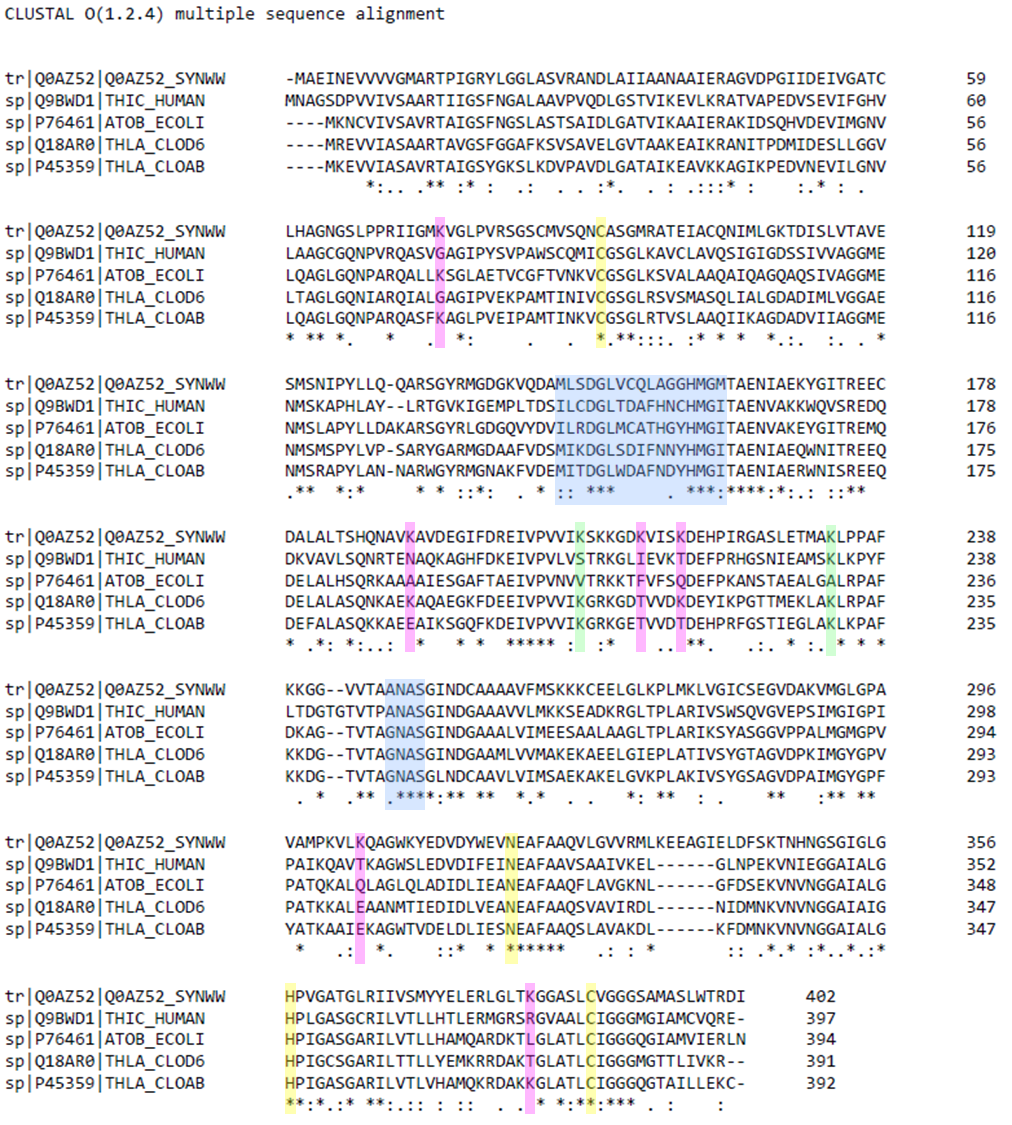


**Supplemental Figure S7.** **CLUSTAL Sequence alignment of acetyl-CoA transferases from model organisms to** ***S. wolfei* Göttingen*.*** Alignment with *S. wolfei* Göttingen Swol_0675, *Homo sapiens* ACAT2 (Q9BWD1), and *Escherichia coli* atoB (P76461), *Clostridium difficile* (Q18AR0), and *C. acetobutylicum* (P45359). Catalytic residues from the four active site loops are highlighted in yellow; covering loop and pantetheine loop are highlighted in blue. Acylated residues identified in *S. wolfei* Göttingen are highlighted in magenta; conserved sites of acylation between *S. wolfei* Göttingen and *C. acetobutylicum* are marked green. Symbols are (:) identical and (*) similar amino acids.

**
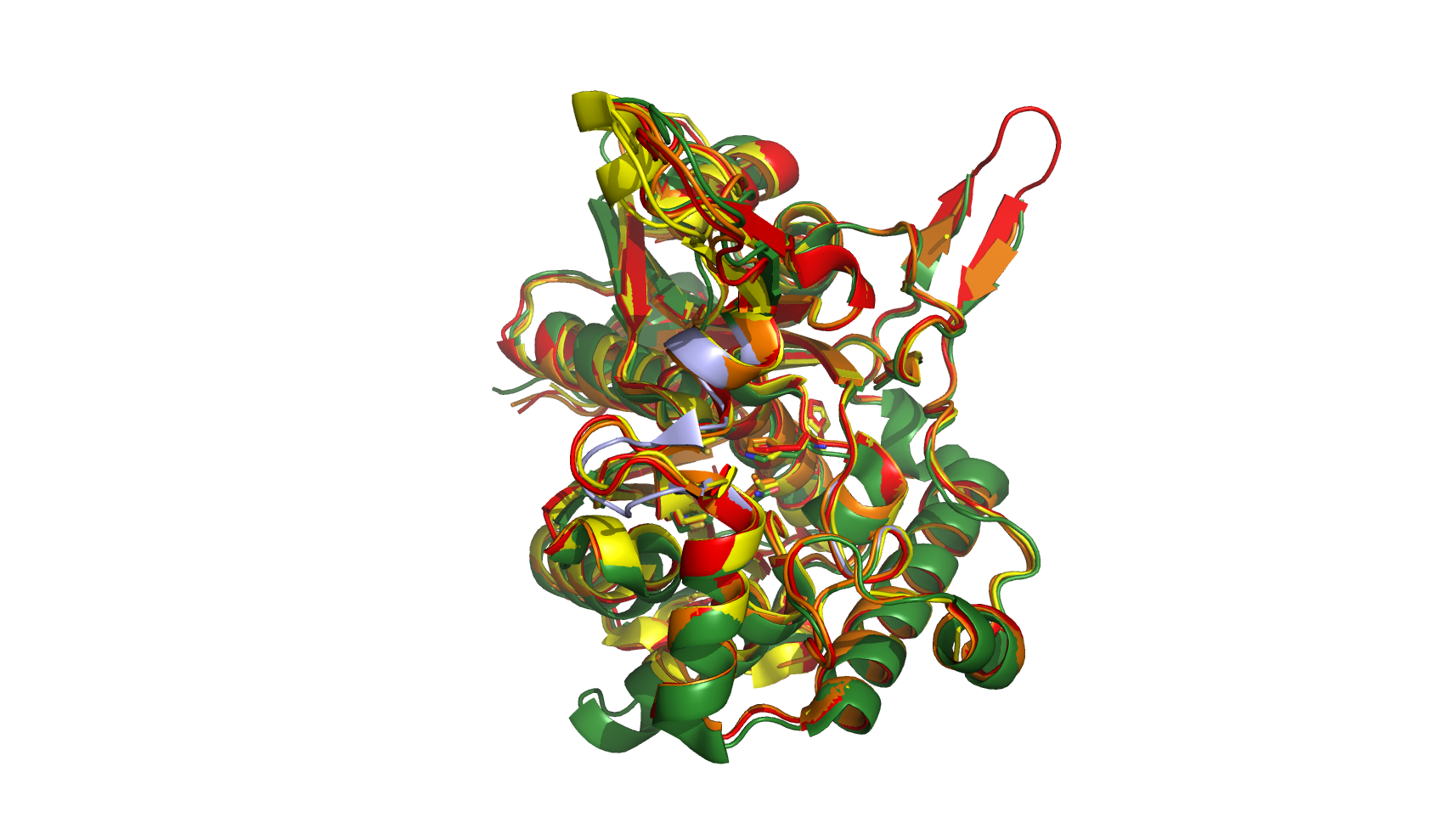
**

**B**

**A**

**
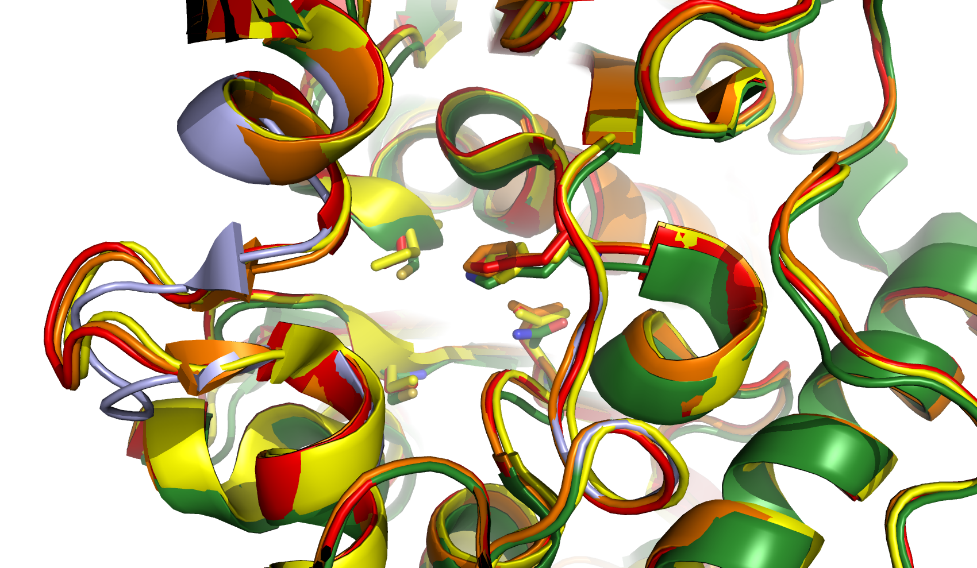
**

**Supplemental Figure S8. Overlay of acetyl-CoA acetyltransferase structures from** ***S. wolfei* Göttingen and other related homologs in Gram-positive and Gram-negative bacteria.** Structural alignment of Act structures from *S. wolfei* Göttingen (green; PDBid 7N7Z), *E. coli* (yellow; PDBid 5F38), *Clostridium difficile* (orange; PDBid 4DD5), and *C. acetobutylicum* (red; PDBid 4WYR). Catalytic loops are in light blue. **(A)** Overall structure of bacterial Acts. **(B)** Active site of Acts.
